# Dengue virus infection in people residing in Africa: a systematic review and meta-analysis of prevalence studies

**DOI:** 10.1038/s41598-019-50135-x

**Published:** 2019-09-20

**Authors:** Fredy Brice N. Simo, Jean Joel Bigna, Sebastien Kenmoe, Marie S. Ndangang, Elvis Temfack, Paul F. Moundipa, Maurice Demanou

**Affiliations:** 1 Reference Laboratory for Chikungunya and Dengue Viruses, Department of Virology, Centre Pasteur of Cameroon, Yaoundé, Cameroon; 2 Department of Biochemistry, Faculty of Sciences, University of Yaoundé I, Yaoundé, Cameroon; 3 Department of Epidemiology and Public Health, Centre Pasteur of Cameroon, Yaoundé, Cameroon; 4 Department of Medical Information and Informatics, Rouen University Hospital, Rouen, France; 5 Department of Internal Medicine, Douala General Hospital, Douala, Cameroon

**Keywords:** Dengue virus, Viral infection, Epidemiology

## Abstract

Better knowledge of the face of the current dengue virus (DENV) epidemiology in Africa can help to implement efficient strategies to curb the burden of dengue fever. We conducted this systematic review and meta-analysis to determine the prevalence of DENV infection in Africa. We searched PubMed, EMBASE, African Journals Online, and Africa Index Medicus from January 1^st^, 2000 to June 10^th^, 2019 without any language restriction. We used a random-effects model to pool studies. A total of 76 studies (80,977 participants; 24 countries) were included. No study had high risk of bias. Twenty-two (29%) had moderate and 54 (71%) had low risk of bias. In apparently healthy individuals, the pooled prevalence of DENV was 15.6% (95% confidence interval 9.9–22.2), 3.5% (0.8–7.8), and 0.0% (0.0–0.5) respectively for immunoglobulins (Ig) G, IgM, and for ribonucleic acid (RNA) in apparently healthy populations. In populations presenting with fever, the prevalence was 24.8% (13.8–37.8), 10.8% (3.8–20.6k) and 8.4% (3.7–14.4) for IgG, IgM, and for RNA respectively. There was heterogeneity in the distribution between different regions of Africa. The prevalence of DENV infection is high in the African continent. Dengue fever therefore deserves more attention from healthcare workers, researchers, and health policy makers.

## Introduction

The incidence of dengue fever has grown in the last decade worldwide. According to the World Health Organization (WHO), 3.9 billion people in 128 countries are at risk of infection and about 390 million of dengue infection occur each year in people worldwide among which 96 million (25%) clinically manifest the disease^1^. DENV is one of the most common arboviruses. It is a tropical and subtropical mosquito bone-viral disease^2,3^. DENV is transmitted by two main epidemic vectors: *Aedes aegypti* and *Aedes albopictus*. These vectors have become widely distributed across tropical and subtropical region and spread globally with the advent of global phenomena such as urbanization, high rate of population growth, inadequate water supply, sewer, poor waste management system, and international travel^2,3^. DENV infection is asymptomatic in more than 50% of cases or can be presented as a flu-like illness including headache, myalgia, and rash like other endemic fever diseases in Africa (example: malaria and chikungunya fever)^2,4^. Currently, there is no treatment for dengue infection. The main method of controlling DENV transmission is through the active monitoring and surveillance of vectors^5,6^.

Before 1970, only 9 countries had experienced severe dengue epidemics. Dengue fever is now endemic in more than 100 countries in the regions of Africa, the Eastern Mediterranean, South-East Asia, the Western Pacific, the Americas. The three last ones are the most seriously affected regions^7^. In Africa, the first reported dengue fever outbreaks occurred in Zanzibar in 1823 and 1870. Several other African countries including Burkina-Faso, Egypt, South Africa and Senegal, reported unconfirmed outbreaks of dengue fever in early 1900s. Although many outbreaks aren’t ever officially reported, between 1960 and 2017, more than 20 laboratory-confirmed dengue epidemics were reported in more than 20 African countries^2,8,9^. A systematic review without meta-analysis by Humphrey and colleagues conducted for Middle East and North Africa have shown a median seroprevalence of dengue infection in general population of 25% (range from 0 to 62%) with most of the seroprevalence studies reported from Red Sea region of Pakistan^9^. Although a total of 22 countries in Africa reported sporadic cases or outbreaks of dengue fever^2^, to date there is no study accurately investigating the epidemiology of DENV infection among febrile and apparently healthy populations in this continent. In order to fill this gap, we conducted this systematic review and meta-analysis with the aim to determine the prevalence of DENV infection in Africa. This review would help better characterize the epidemiology in the continent and build effective interventions to curb the burden of dengue fever in the continent.

## Methods

### Design

The Preferred Reporting Items for Systematic Reviews and Meta-analyses guidelines served as the template for reporting the present review (Supplementary Table 1, Appendix). This review is registered in the PROSPERO International Prospective Register of Systematic Reviews, registration number CRD42018104829. For this systematic review and meta-analysis, we used the same methodology as in already published meta-analysis of prevalence studies for chikungunya fever^10^.

### Criteria for considering studies for the review

We considered observational studies (cross-sectional, case-control, and cohort). Studies in which DENV infection were imported were excluded. Studies had to be conducted out of outbreak periods. DENV infection had to be diagnosed by the detection of the three biological markers of DENV including RNA, Immunoglobulins (Ig) M, or G; among people residing in Africa. Studies lacking or with no extractable primary data, and/or explicit methods description, were excluded. In the case of missing data, we contacted authors.

### Strategy to identify relevant studies

A comprehensive search of MEDLINE through PubMed, Excerpta Medica database (EMBASE), African Journals Online, and Africa Index Medicus was conducted to identify all relevant articles published on DENV infection among people living in Africa from January 1^st^, 2000 to June 10^th^, 2019, without any language restriction. We considered recent studies to have current and updated overview on the epidemiology in the continent. A search strategy based on the combination of relevant terms was designed and applied. The main search strategy in EMBASE is shown in Supplementary Table 2 (Appendix). This search strategy has been adapted for search in other databases. A manual search that consists of scanning reference lists of eligible studies and relevant reviews was performed.

### Selection of studies to include in the review

Two investigators independently screened records for eligibility based on titles and abstracts. Full-texts of articles deemed potentially eligible were retrieved. Further, these investigators independently assessed the full-text of each study for eligibility, and consensually retained studies to be included. Disagreements were solved through a discussion or by an arbitration of a third investigator.

### Data extraction and management

The data have been extracted using a preconceived, piloted and standardized data abstraction form. Two investigators independently extracted data including: name of first author, year of publication, title of the study; recruitment site (country, city), setting (rural, urban), sampling method; number of persons tested for DENV, number of persons infected with DENVs, mean and median age, proportion male, and clinical presentation. In the case of multinational studies, data have been separated to present the estimate in each country when possible. We assigned a United Nations Statistics Division (UNSD) African region (Central, Eastern, Northern, Southern, and Western) to each study according to the country of recruitment^11^. We have also identified studies where plaque resorption neutralization test (PRNT) where used to measure specific neutralizing antibodies titers helping to rule out false-positive ELISA results^12^.

### Methodological quality assessment

We used an adapted version of the tool developed by Hoy and colleagues to assess the methodological quality of included studies^13^. Two investigators independently ran the assessment. Discrepancies were discussed and resolved by these investigators. Inter-rater agreements between investigators for study inclusion and methodological quality assessment were assessed using Cohen’s κ.

### Data synthesis and analysis

Data have been analyzed using the ‘*meta*’ packages of the R statistical software (version 3.5.1, The R Foundation for statistical computing, Vienna, Austria). We used the reference method to synthetize prevalence data as recommended by Barendregt and colleagues^14^. The prevalence of DENV infection has been recalculated on the basis of crude numerators and denominators provided by individual studies. To minimize the effect of studies with extremely small or extremely large prevalence estimates on the overall estimate, the variance of study-specific prevalence was stabilized with the Freeman-Tukey double arcsine transformation before pooling the data with the random effects meta-analysis model^14^. Heterogeneity was assessed by the chi-square test on Cochrane’s Q statistic^15^, which was quantified by I-squares values, assuming I-squares values of 25, 50 and 75% respectively representing low, medium and high heterogeneity^16^. When substantial heterogeneity was detected (I² > 50%), we performed subgroup analyzes to investigate possible sources of heterogeneity using the following grouping variables: clinical presentation (febrile vs apparently healthy) and UNSD regions. The symmetry of the funnel plots and the Egger test have been used to assess the presence of publication bias^17^. A *p* value < 0.10 was considered indicative of a statistically significant publication bias for studies reporting the prevalence of dengue fever^18^. It was decided a priori that if publication bias were present it would not be adjusted for, since we believed that the prevalence estimates of interest would likely be published even if substantially different from previously reported estimates. In the case of multinational studies, we considered data from each country as one study before the meta-analysis. This was also done in cases of studies including both febrile and non-febrile patients included in the same study. To assess the robustness of our findings, we conducted a sensitivity analysis considering only studies that used a confirmation analysis by PRNT.

## Results

### Study selection and characteristics

**Figure 1:**
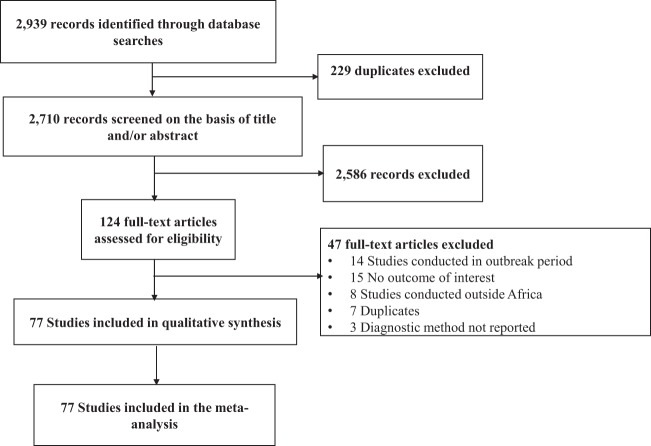
Study selection process.

In total, we identified 2,554 records, of which 77 studies were included (Fig. 1). The final list of included studies is in the Appendix. Agreement between review authors for study selection based on title and abstract (κ = 0.86) and data extraction (κ = 0.78) were moderate to high. No study had high risk of bias. Twenty-two (29%) had moderate and 54 (71%) had low risk of bias. Twenty-six and 50 studies were conducted in apparently healthy and febrile participants respectively. Sixty-four and 12 studies were prospective and retrospective respectively. All studies were cross-sectional and conducted from 2000 to 2017. Proportion of males varied from 0% to 97.2% (61 studies). Data were only from sub-Saharan Africa. In total, 38 studies were from Eastern, 27 from Western, 10 from Central, and 1 from Southern Africa. Data were from 23 countries: Angola (n = 1), Burkina-Faso (n = 2), Cameroon (n = 3), Comoros (n = 1), Côte d’Ivoire (n = 4), Democratic Republic of the Congo (n = 1), Djibouti (n = 3), Ethiopia (n = 3), Gabon (n = 4), Ghana (n = 3), Guinea (n = 1), Kenya (n = 8), Mali (n = 1), Mayotte (n = 1), Namibia (n = 1), Nigeria (n = 12), Sao Tome and Principe (n = 1), Senegal (n = 1), Sierra Leone (n = 3), South Sudan (n = 1), Sudan (n = 10), Tanzania (n = 9), Zambia (n = 1), and Zimbabwe (n = 1). Mean/median age of participants varied from 1.2 to 47.9 years (38 studies). Individual characteristics of included studies are in the Supplementary Table 3 (Appendix).

### Prevalence of dengue virus infection in africa

**Figure 2:**
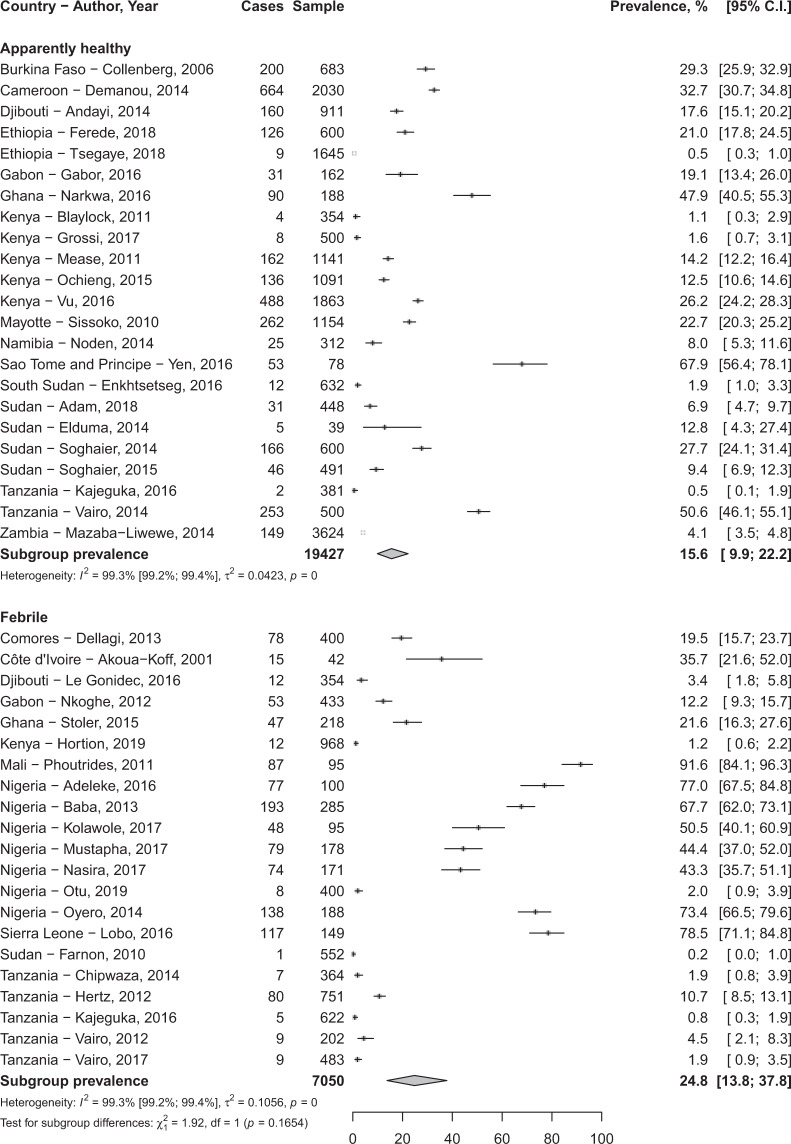
Meta-analysis of immunoglobulins G seroprevalence of Dengue virus in Africa.

In total, 80,977 participants were included in the 76 studies. The prevalence of DENV infection varied widely depending on clinical presentation and biomarkers of infection considered (Table 1). The IgG seroprevalence in febrile participants was 24.8% (95%CI 13.8–37.8) and 15.6% (95%CI 9.9–22.2) in apparently healthy individuals without significant difference (*p* = 0.165) (Fig. 2; Table 1). Sensitivity analysis considering only study with DENV infection confirmation by PNRT test yielded findings in the range of that of crude analysis (Table 1).

**Table 1:** Meta-analysis prevalence of dengue infection in febrile and apparently healthy populations in Africa.

	Prevalence, % (95% confidence interval)	N studies	N participants	I² (95% confidence interval)	H (95% confidence interval)	*p* heterogeneity	*p* Egger test	*p* difference
**Crude analysis**								
IgG seroprevalence								
In febrile	24.8 (13.8–37.8)	21	7050	99.3 (99.2–99.4)	11.8 (10.0–12.7)	<0.0001	0.0001	0.165
In apparently healthy	15.6 (9.9–22.2)	24	19427	99.3 (99.2–99.4)	11.8 (11.0–12.6)	<0.0001	0.455	
IgM seroprevalence								
In febrile	10.8 (3.8–20.6)	36	40251	99.8 (99.8–99.8)	24.8 (24.0–25.6)	<0.0001	0.822	0.084
In apparently healthy	3.5 (0.8–7.7)	8	14942	98.4 (97.8–98.8)	7.9 (6.7–9.2)	<0.0001	0.197	
Detected RNA								
In febrile	7.1 (3.3–12.0)	21	15322	99.0 (98.8–99.1)	9.9 (9.1–10.7)	<0.0001	0.362	<0.0001
In apparently healthy	0.0 (0.0–0.5)	1	188	NA	NA	NA	NA	
Sensitivity analysis*								
IgG seroprevalence								
In febrile	19.4 (4.1–41.9)	8	3081	99.4 (99.3–99.6)	13.4 (12.0–15.0)	<0.0001	0.104	0.940
In apparently healthy	18.2 (9.5–28.9)	11	14854	99.6 (99.5–99.6)	15.1 (13.8–16.4)	<0.0001	0.233	
IgM seroprevalence								
In febrile	3.1 (0.0–20.2)	11	32259	100 (99.0–100)	45.0 (43.3–46.7)	<0.0001	0.592	0.330
In apparently healthy	0.1 (0.0–0.4)	1	2030	NA	NA	NA	NA	

^*^Only studies with confirmation of dengue virus infection by plaque reduction neutralization test.

**Figure 3:**
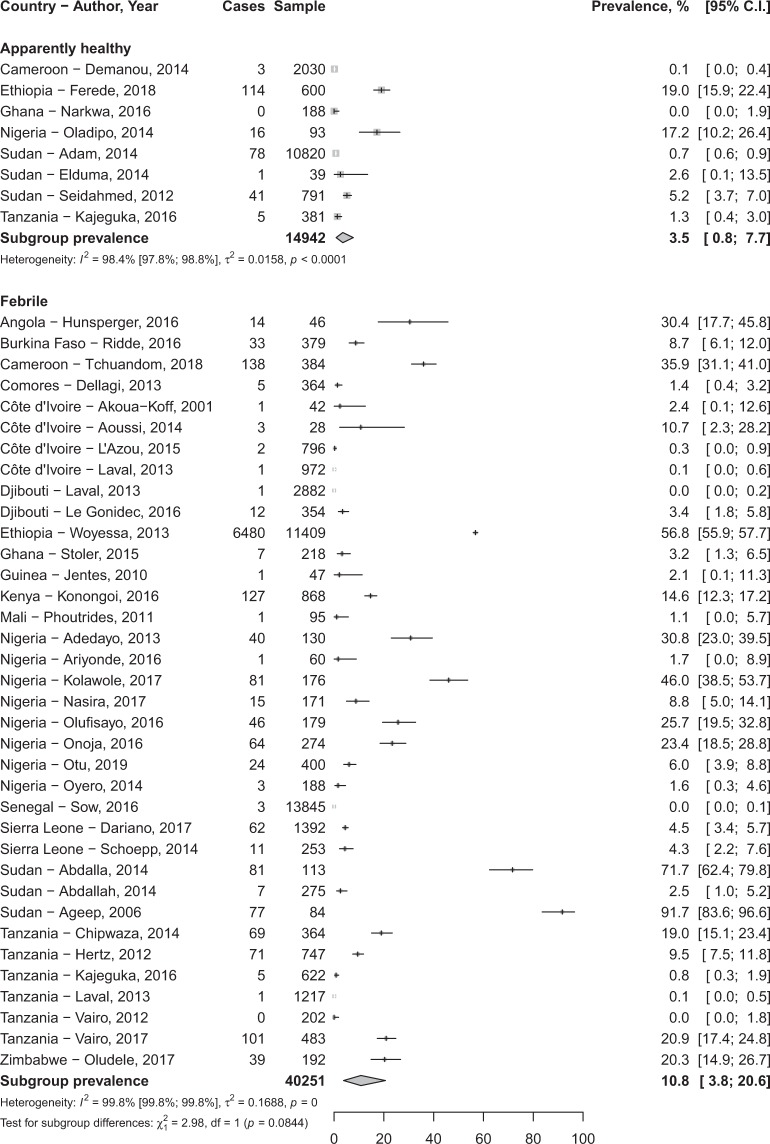
Meta-analysis of immunoglobulins M seroprevalence of Dengue virus in Africa.

The prevalence of IgM seroprevalence in febrile participants 10.8% (95%CI 3.8–20.6) was higher compared to apparently healthy participants 3.5% (95%CI 0.8–7.8) without significant difference, *p* = 0.084 (Fig. 3; Table 1).

**Figure 4:**
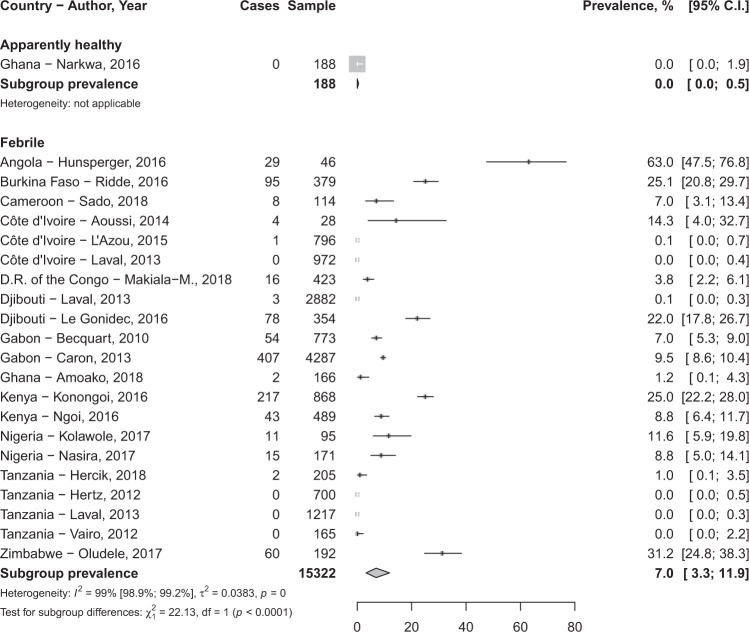
Meta-analysis prevalence of Dengue virus ribonucleic acid in Africa.

The DENV RNA prevalence in febrile participants 8.4% (95%CI 3.7–14.4) was significantly higher compared to apparently healthy participants 0.0% (95%CI 0.0–0.5), *p* < 0.0001 (Fig. 4; Table 1).

We found substantial heterogeneity for all meta-analyses (Table 1). Publication bias was detected for IgG seroprevalence in febrile participants (*p* = 0.0007, Table 1) as depicted in the funnel plot (Supplementary Fig. 1, Appendix). There was no publication bias for other analyses (Table 1 and Supplementary Figs 1–5, Appendix).

### Prevalence of dengue virus infection in sub-regions of africa

There was a wide variation of DENV infection prevalence between sub-regions in Africa. There was difference in the DENV infection IgG seroprevalence between regions for participants with fever, varying from 3.6% in Eastern Africa to 52.6% in Western Africa, *p* < 0.0001 (no data from Southern Africa; Supplementary Fig. 6). There was a difference in the DENV infection IgG seroprevalence between regions in apparently healthy participants, varying from 8.0% in Southern Africa to 38.6% in Central Africa, *p* < 0.0001 (Supplementary Fig. 7). The IgM seroprevalence in febrile participants varied from 6.6% in Western Africa to 35.2% in Central Africa, *p* < 0.001 (Supplementary Fig. 8). Among apparently healthy participants, this prevalence varied from 0.1% in Central Africa to 5.0% in Eastern Africa, *p* = 0.044 (Supplementary Fig. 9). For RNA prevalence in febrile participants, the prevalence varied from 5.3% in Eastern Africa to 12.2% in Central Africa, without significant difference, *p* = 0.270 (Supplementary Fig. 10). There were not enough studies to perform subgroup analysis for RNA prevalence among apparently healthy participants.

## Discussion

This first systematic review with meta-analysis of the prevalence of DENV infection among people residing in Africa depicted a relatively high prevalence with substantial heterogeneity depending on clinical presentation, viral markers considered, and geographical distribution.

The seroprevalence obtained in this study is in the range of that found in the review by Humphrey and colleagues conducted in urban areas of Middle East and North Africa, with majority of the studies from Red Sea region^9^. Humphrey and colleagues reported a seroprevalence in general population of 25% ranging from 0 to 62% in studies conducted from 1941 to 2015^9^. However, this previous review did not differentiate type of seroprevalence between IgG and IgM. In addition, RNA of DENV was not considered.

The high prevalence we found in this review may reflect the weakness of the ongoing public health interventions to control vector transmission of DENV. This can be explained by weak health systems in a low-income context leading to inability to sustain efficient and continuously powerful interventions. In addition, in certain African countries any strategies were implemented to date for vector control^5,6,9^. The increasing rate of urbanization in the continent can also explain the high DENV infection prevalence. Indeed, high urbanization rate with poor infrastructure in tropical and subtropical regions is associated with high burden of DENV infection^2,3^. The mechanism would be the increase of mosquitoes breeding sites in these developing countries. The ecological environment and climatological patterns of tropical and subtropical regions where most of African countries are located also favor vector long-life and development^19,20^. Indeed, in warm and humid climate that favors eggs conservation and development, *Aedes albopictu*s and *Aedes aegypti* are active all year long compared to cooler, temperate regions, where they hibernate over winter^21–24^. Another explanation and one of the most important for the high prevalence is the interaction between all factors evocated above in addition to the facilitation of international travelling due to development of transportation that favor the expansion of dengue vectors in Africa^25–28^. One of DENV vectors, *Aedes albopictus*, originally came from tropical and subtropical region of Southeast Asia. The presence of this vector in Africa has been facilitated by the development of international transport. For example, the tire trade between Asia, Europe, and Africa was the most favorable factor for the transfer of mosquitoes eggs from Asia or Europe to Africa^29–31^. Definitively, in the last decades there is an increasing urbanization, increasing human exchange between countries and ecological imbalance favoring the infection of vectors, and then of other never infected humans.

We found a higher prevalence with IgG compared to IgM and RNA markers. RNA is detectable in patients only from infection up to six days after the onset of the fever. IgM are detectable five days after the onset of fever up to three months while IgG appear on the tenth day after the onset of fever and can persist for several years^3^. The prevalence was not surprisingly higher in febrile compared to apparently healthy individuals, although not significant. This may be explained by the fact that the viral markers are more prevalent during febrile phase (first seven days). We found a higher prevalence of IgG in the febrile patients compared to apparently healthy. As IgG can stay several years in the serum, an episode of fever can occur in an individual who already had a previous infection (with IgG and IgM production), especially in the endemic context for DENV like Africa. During a new acute infection (a second and others after), the secondary immunological response is faster with a higher IgG production^32^.

There was also a heterogeneity in prevalence according to geographical areas. Since not all regions and countries were represented, this finding needs a cautious interpretation. In addition, the UNSD of the Africa continent is only an administrative subdivision and could not efficiently map the epidemiology. We found that western Africa had the highest IgG seroprevalence in febrile participants and the highest IgM seroprevalence in apparently healthy individuals, that western and central Africa had the highest IgG seroprevalence in apparently healthy individuals, and that central Africa had the highest IgM seroprevalence and RNA prevalence in febrile participants. Higher prevalence in both western and central Africa may be the reflection of weak quality of infrastructures in urban areas in these regions, weaker healthcare systems, and optimal ecological environment and climatological patterns for lifecycle of DENV. However, studies are needed to deeply investigate the heterogeneity of epidemiology of DENV infection between and within countries of the continent. It would be better to have the geospatial distribution of DENV vectors in the continent to better understand the distribution of DENV infection among humans. Heterogeneity in the prevalence of dengue infection in Africa may also be due to the difference of the immunity of human across the continent and the heterogeneity of vectors distribution^33–35^. In addition, in some African countries, both epidemic vectors *Aedes aegypti* and *Aedes albopictus* are found and only *Aedes aegypti* in other countries^34,36^.

The findings of this review have important inference for clinical practice, researchers, and health policies for the continent. To date, there is no effective antiviral treatment for dengue fever^7^. With the high prevalence we found, especially among patients presenting with fever, implementation of routine diagnostic for people living in endemic areas or for people presenting fever with history of a travel in dengue endemic area may be explored^37,38^. This can be motivated by the fact that, proper medical management of early detected can lower fatality rates below 1% and there is no specific treatment against DENV infection^7^. It would be also interesting to explore the implementation of routine diagnostic of DENV infection in endemic countries with the aim to make differential diagnosis with other infectious diseases (like malaria) with close clinical presentation. However, for endemic areas with most of them locating in developing countries like in Africa, it will be difficult to implement routine diagnostic of DENV infection at a large scale as most of countries have weak health systems with low funding. Our findings call for cost-effective strategies to curb the burden DENV infection in Africa since severe dengue fever can rapidly lead to serious illness and death, especially among children^7^. To date, the efficacy of dengue vaccine has been accessed in eleven countries with immune response of 59% of vaccinated people^39^, suggesting that high quality studies are needed to have highly effective vaccine and antiretroviral treatment to rapidly curb the burden of dengue fever in Africa. Another approach for curbing the burden of dengue fever is vector control. For curbing the burden of vector-borne disease, WHO suggests an integrated vector control. This vector control should be environmentally safe when using insecticides for the elimination of mosquitoes Laval from their artificial and natural habitats. This is to ensure the protection of individuals, communities, and households in an ecological context to be effective and efficient^10,40^.

This review has some limitations. They may be a heterogeneity between diagnostic tools used in different included studies. Other sources of heterogeneity not explored in this study, because they are not reported in primary studies, include the difference in the prevalence of DENV in vectors, the difference in the immunity of humans, the difference in serotypes and genotypes of DENV, and ecosystem of different study places across the continent. All countries were not represented in this systematic review. This can hinder the generalization of our findings. Although there were some limitations, this is to the very best of our knowledge, the first review with meta-analysis summarizing data on the epidemiology of DENV infection in febrile and apparently healthy populations living in Africa. We have performed a comprehensive search of the literature and included independent reviewers for study selection and data extraction to avoid biases. Almost three quarters of the included studies were assessed as having low risk of bias. This suggest that we can be confident in the quality of findings this review. In addition, our main findings were in the range to that of sensitivity analysis considering only studies with PNRT for DENV confirmation; highlighting the robustness of our findings.

We can definitely conclude that the prevalence of DENV infection is high in the African continent although this infectious disease is considered as neglected tropical disease. It is therefore necessary that dengue fever deserves more attention from healthcare workers, researchers, and health policy makers in the continent. In the absence to date of effective antiviral treatment for dengue fever, strategies to curb its burden should therefore focus on prevention including vector control.

## Supplementary information

Appendix

## Data Availability

All data generated or analyzed during this study are included in this published article and its supplementary information files.
