# Supplementary material for: Dengue virus infection in people residing in Africa: a systematic review and meta-analysis of prevalence studies
Source: Sci Rep. 2019 Sep 20;9:13626. doi: 10.1038/s41598-019-50135-x (PMC6754462; doi:10.1038/s41598-019-50135-x)
Supplement: Supplementary file 1 — Appendix [file 41598_2019_50135_MOESM1_ESM.pdf]

Dengue virus infection in people residing in Africa: a systematic review and meta-analysis of prevalence studies

Fredy Brice N. **Simo,** Jean Joel **Bigna,** Sebastien **Kenmoe,** Marie S. **Ndangang,**
Elvis **Temfack,** Paul F. **Moundipa,** Maurice **Demanou**

Appendix

Supplementary Tables

Supplementary Table 1 : PRISMA checklist 2
Supplementary Table 2 : Search strategy in EMBASE 5
Supplementary Table 3. Risk of bias tool to assess methodological quality of included studies 6
Supplementary Table 4 : Individual characteristics of included studies 7

Supplementary figures

Supplementary Figure 1 : Funnel plot for publication in the IgG seroprevalence of Dengue virus infection in febrile participants in Africa 10
Supplementary Figure 2 : Funnel plot for publication in the IgG seroprevalence of Dengue virus infection in apparently healthy participants in Africa 10
Supplementary Figure 3 : Funnel plot for publication in the IgM seroprevalence of Dengue virus infection in febrile participants in Africa 11
Supplementary Figure 4 : Funnel plot for publication in the IgM seroprevalence of Dengue virus infection in apparently healthy participants in Africa 11
Supplementary Figure 5 : Funnel plot for publication in the RNA prevalence of Dengue virus infection in febrile participants in Africa 12
Supplementary Figure 6 : Forest plot IgG seroprevalence of Dengue virus infection in febrile participants in Africa, by region 13
Supplementary Figure 7 : Forest plot IgG seroprevalence of Dengue virus infection in apparently healthy participants in Africa, by region 14
Supplementary Figure 8 : Forest plot IgM seroprevalence of Dengue virus infection in febrile participants in Africa, by region 15
Supplementary Figure 9 : Forest plot IgM seroprevalence of Dengue virus infection in apparently healthy participants in Africa, by region 16
Supplementary Figure 10 : Forest plot dengue virus RNA prevalence of Dengue virus infection in febrile participants in Africa, by region 17

Supplementary Table 1 : PRISMA checklist

Section/topic	#	Checklist item	Reported on page #
**TITLE**			
Title	1	Identify the report as a systematic review, meta-analysis, or both.	1
**ABSTRACT**			
Structured summary	2	Provide a structured summary including, as applicable: background; objectives; data sources; study eligibility criteria, participants, and interventions; study appraisal and synthesis methods; results; limitations; conclusions and implications of key findings; systematic review registration number.	2
**INTRODUCTION**			
Rationale	3	Describe the rationale for the review in the context of what is already known.	3
Objectives	4	Provide an explicit statement of questions being addressed with reference to participants, interventions, comparisons, outcomes, and study design (PICOS).	3-4
**METHODS**			
Protocol and registration	5	Indicate if a review protocol exists, if and where it can be accessed (e.g., Web address), and, if available, provide registration information including registration number.	4
Eligibility criteria	6	Specify study characteristics (e.g., PICOS, length of follow-up) and report characteristics (e.g., years considered, language, publication status) used as criteria for eligibility, giving rationale.	4
Information sources	7	Describe all information sources (e.g., databases with dates of coverage, contact with study authors to identify additional studies) in the search and date last searched.	4-5
Search	8	Present full electronic search strategy for at least one database, including any limits used, such that it could be repeated.	4-5
Study selection	9	State the process for selecting studies (i.e., screening, eligibility, included in systematic review, and, if applicable, included in the meta-analysis).	5
Data collection process	10	Describe method of data extraction from reports (e.g., piloted forms, independently, in duplicate) and any processes for obtaining and confirming data from investigators.	5
Data items	11	List and define all variables for which data were sought (e.g., PICOS, funding sources) and any assumptions and simplifications made.	5

Risk of bias in individual studies	12	Describe methods used for assessing risk of bias of individual studies (including specification of whether this was done at the study or outcome level), and how this information is to be used in any data synthesis.	5-6
Summary measures	13	State the principal summary measures (e.g., risk ratio, difference in means).	6
Synthesis of results	14	Describe the methods of handling data and combining results of studies, if done, including measures of consistency (e.g., I^2) for each meta-analysis.	6
Risk of bias across studies	15	Specify any assessment of risk of bias that may affect the cumulative evidence (e.g., publication bias, selective reporting within studies).	6
Additional analyses	16	Describe methods of additional analyses (e.g., sensitivity or subgroup analyses, meta-regression), if done, indicating which were pre-specified.	6-7
**RESULTS**			
Study selection	17	Give numbers of studies screened, assessed for eligibility, and included in the review, with reasons for exclusions at each stage, ideally with a flow diagram.	7
Study characteristics	18	For each study, present characteristics for which data were extracted (e.g., study size, PICOS, follow-up period) and provide the citations.	7
Risk of bias within studies	19	Present data on risk of bias of each study and, if available, any outcome level assessment (see item 12).	7
Results of individual studies	20	For all outcomes considered (benefits or harms), present, for each study: (a) simple summary data for each intervention group (b) effect estimates and confidence intervals, ideally with a forest plot.	8
Synthesis of results	21	Present results of each meta-analysis done, including confidence intervals and measures of consistency.	8
Risk of bias across studies	22	Present results of any assessment of risk of bias across studies (see Item 15).	8
Additional analysis	23	Give results of additional analyses, if done (e.g., sensitivity or subgroup analyses, meta-regression [see Item 16]).	9
**DISCUSSION**			
Summary of evidence	24	Summarize the main findings including the strength of evidence for each main outcome; consider their relevance to key groups (e.g., healthcare providers, users, and policy makers).	9
Limitations	25	Discuss limitations at study and outcome level (e.g., risk of bias), and at review-level (e.g., incomplete retrieval of identified research, reporting bias).	12
Conclusions	26	Provide a general interpretation of the results in the context of other evidence, and implications for future research.	13
**FUNDING**			

Funding	27	Describe sources of funding for the systematic review and other support (e.g., supply of data); role of funders for the systematic review.	13

From: Moher D, Liberati A, Tetzlaff J, Altman DG, The PRISMA Group (2009). Preferred Reporting Items for Systematic Reviews and Meta-Analyses: The PRISMA Statement. PLoS Med 6(6): e1000097. doi:10.1371/journal.pmed1000097

Supplementary Table 2 : Search strategy in EMBASE

Search	Terms
#1	“dengue” OR "dengue virus" OR “breakbone fever” OR “break-bone fever”
#2	africa* OR 'algeria'/exp OR algeria OR 'angola'/exp OR angola OR 'benin'/exp OR benin OR 'botswana'/exp OR botswana OR 'burkina faso'/exp OR 'burkina faso' OR 'burundi'/exp OR burundi OR 'cameroon'/exp OR cameroon OR 'canary islands'/exp OR 'canary islands' OR 'cape verde'/exp OR 'cape verde' OR 'central african republic'/exp OR 'central african republic' OR 'chad'/exp OR chad OR 'comoros'/exp OR comoros OR 'congo'/exp OR congo OR 'democratic republic of congo' OR 'djibouti'/exp OR djibouti OR 'egypt'/exp OR egypt OR 'equatorial guinea'/exp OR 'equatorial guinea' OR 'eritrea'/exp OR eritrea OR 'ethiopia'/exp OR ethiopia OR 'gabon'/exp OR gabon OR 'gambia'/exp OR gambia OR 'ghana'/exp OR ghana OR 'guinea'/exp OR guinea OR 'guinea bissau'/exp OR 'guinea bissau' OR 'ivory coast'/exp OR 'ivory coast' OR 'cote ivoire' OR jamahiriya OR 'kenya'/exp OR kenya OR 'lesotho'/exp OR lesotho OR 'liberia'/exp OR liberia OR 'libya'/exp OR libya OR 'madagascar'/exp OR madagascar OR 'malawi'/exp OR malawi OR 'mali'/exp OR mali OR 'mauritania'/exp OR mauritania OR 'mauritius'/exp OR mauritius OR 'mayotte'/exp OR mayotte OR 'morocco'/exp OR morocco OR 'mozambique'/exp OR mozambique OR 'namibia'/exp OR namibia OR 'niger'/exp OR niger OR 'nigeria'/exp OR nigeria OR principe OR 'reunion'/exp OR reunion OR 'rwanda'/exp OR rwanda OR 'sao tome' OR 'senegal'/exp OR senegal OR 'seychelles'/exp OR seychelles OR 'sierra leone'/exp OR 'sierra leone' OR 'somalia'/exp OR somalia OR 'south africa'/exp OR 'south africa' OR 'st helena'/exp OR 'st helena' OR 'sudan'/exp OR sudan OR 'swaziland'/exp OR swaziland OR 'tanzania'/exp OR tanzania OR 'togo'/exp OR togo OR 'tunisia'/exp OR tunisia OR 'uganda'/exp OR uganda OR 'western sahara'/exp OR 'western sahara' OR 'zaire'/exp OR zaire OR 'zambia'/exp OR zambia OR 'zimbabwe'/exp OR zimbabwe OR 'central africa'/exp OR 'central africa' OR 'central african'/exp OR 'central african' OR 'west africa'/exp OR 'west africa' OR 'west african'/exp OR 'west african' OR 'western africa'/exp OR 'western africa' OR 'western african'/exp OR 'western african' OR 'east africa'/exp OR 'east africa' OR 'east african'/exp OR 'east african' OR 'eastern africa'/exp OR 'eastern africa' OR 'eastern african'/exp OR 'eastern african' OR 'north africa'/exp OR 'north africa' OR 'north african'/exp OR 'north african' OR 'northern africa'/exp OR 'northern africa' OR 'northern african'/exp OR 'northern african' OR 'south african'/exp OR 'south african' OR 'southern africa'/exp OR 'southern africa' OR 'southern african'/exp OR 'southern african' OR 'sub saharan africa'/exp OR 'sub saharan africa' OR 'sub saharan african' OR 'subsaharan africa'/exp OR 'subsaharan africa' OR 'subsaharan african'
#3	#1 AND #2
#4	[2000-2019]/py
#5	#3 AND #4

Supplementary Table 3. Risk of bias tool to assess methodological quality of included studies

Risk of bias items
Was the study's target population a close representation of the national population in relation to relevant variables?
Was the sampling frame a true or close representation of the target population?
Was some form of random selection used to select the sample, OR was a census undertaken?
Was the likelihood of nonresponse bias minimal?
Were data collected directly from the subjects (as opposed to a proxy)?
Was an acceptable case definition used in the study?
Was the study instrument that measured the parameter of interest shown to have validity and reliability?
Was the same mode of data collection used for all subjects?
Were the numerator(s) and denominator(s) for the parameter of interest appropriate?

0-3 items: high risk of bias; 4-6: Moderate risk of bias; 7-9: low risk of bias

Supplementary Table 4 : Individual characteristics of included studies

Author	Clinical presentation	Year	#Center	Timing	Design	Period of recruitment	Country	Sample	Rob	Sampling	Sample type
**Abdalla**	Febrile	2014	One site	Retrospective	Cross-sectional	Oct/2010-Nov/2010	Sudan	113	Low	Consecutive	Blood
**Abdallah**	Febrile	2014	One site	Prospective	Cross-sectional	Jan/2012–Mar/2012	Sudan	275	Low	Consecutive	Blood
**Adam**	Apparently healthy	2014	One site	Retrospective	Cross-sectional	2008-2009	Sudan	10820	Low	Consecutive	Blood
**Adam**	Apparently healthy	2018	One site	Prospective	Cross-sectional	Dec/2012-Jan/2013	Sudan	448	Low	Consecutive	Blood
**Adedayo**	Febrile	2013	One site	Prospective	Cross-sectional	Not defined	Nigeria	130	Low	Consecutive	Blood
**Adeleke**	Febrile	2016	One site	Prospective	Cross-sectional	Jul/2014-Sep/2014	Nigeria	100	Low	Consecutive	Blood
**Ageep**	Febrile	2006	One site	Prospective	Cross-sectional	Apr/2005-Jul/2005	Sudan	84	Low	Consecutive	Blood
**Akoua-Koff**	Febrile	2001	One site	Prospective	Cross-sectional	Aug/1999	Côte d'Ivoire	42	Low	Consecutive	Blood
**Amoako**	Febrile	2018	One site	Prospective	Cross-sectional	Oct/2016-Jul/2017	Ghana	700	Moderate	Consecutive	Blood
**Andayi**	Apparently healthy	2014	One site	Prospective	Cross-sectional	Nov/2010-Feb/2011	Djibouti	911	Low	Consecutive	Blood
**Aoussi**	Febrile	2014	One site	Retrospective	Cross-sectional	May/2010-Oct/2010	Côte d'Ivoire	28	Moderate	Consecutive	Blood
**Ariyonde**	Febrile	2016	One site	Prospective	Cross-sectional	Apr/2014-May/2014	Nigeria	60	Low	Consecutive	Blood
**Baba**	Febrile	2013	One site	Prospective	Cross-sectional	Jul/2008-Dec/2008	Nigeria	285	Low	Consecutive	Blood
**Becquart**	Febrile	2010	One site	Prospective	Cross-sectional	Mar2007-Aug2007	Gabon	773	Low	Consecutive	Blood
**Blaylock**	Apparently healthy	2011	One site	Retrospective	Cross-sectional	Not defined	Kenya	354	Moderate	Consecutive	Blood
**Caron**	Febrile	2013	One site	Prospective	Cross-sectional	Sep/2007-Aug/2010	Gabon	4287	Low	Consecutive	Blood
**Chipwaza**	Febrile	2014	One site	Prospective	Cross-sectional	Mar/2013-Oct/2013	Tanzania	364	Moderate	Consecutive	Blood
**Collenberg**	Apparently healthy	2006	One site	Prospective	Cross-sectional	Jul/2003-Jan/2004	Burkina Faso	683	Low	Consecutive	Blood
**Dariano**	Febrile	2017	One site	Prospective	Cross-sectional	Jul/2012-Jun/2013	Sierra Leone	1392	Low	Consecutive	Blood
**Dellagi**	Febrile	2013	One site	Prospective	Cross-sectional	Aug/2011-Oct/2011	Comores	400	Moderate	Consecutive	Blood
**Demanou**	Apparently healthy	2014	One site	Prospective	Cross-sectional	Sep/2006-Dec/2007	Cameroon	2030	Low	Cluster	Blood
**Elduma**	Apparently healthy	2014	One site	Prospective	Cross-sectional	Jan/2012-Apr/2012	Sudan	39	Low	Consecutive	Blood
**Enkhtsetseg**	Apparently healthy	2016	One site	Prospective	Cross-sectional	2012-2013	South Sudan	632	Low	Consecutive	Blood
**Farnon**	Febrile	2010	One site	Prospective	Cross-sectional	Dec/ 2005	Sudan	552	Low	Consecutive	Blood
**Ferede**	Apparently healthy	2018	One site	Prospective	Cross-sectional	Mar/2016-May/2017	Ethiopia	600	Low	Consecutive	Blood
**Gabor**	Apparently healthy	2016	One site	Retrospective	Cross-sectional	Dec/2002-Apr/2007	Gabon	162	Moderate	Clutser Random	Blood

**Grossi**	Apparently healthy	2017	One site	Prospective	Cross-sectional	Aug/2010-Jul/2012	Kenya	500	Low	Stratified Random	Blood
**Hercik**	Febrile	2018	One site	Prospective	Cross-sectional	Jun-Jul/2014	Tanzania	205	Low	Consecutive	Blood
**Hertz**	Febrile	2012	One site	Prospective	Cross-sectional	Sep/2007-Aug/2008	Tanzania	751	Low	Consecutive	Blood
**Hortion**	Febrile	2019	One site	Prospective	Cross-sectional	Dec/2014-Dec/2015	Kenya	968	Low	COnsecutive	Blood
**Hunsperger**	Febrile	2016	One site	Prospective	Cross-sectional	2013	Angola	46	Moderate	Consecutive	Blood
**Jentes**	Febrile	2010	One site	Prospective	Cross-sectional	Aug/2006-Jan/2007	Guinea	47	Low	Consecutive	Blood
**Kajeguka**	Apparently healthy	2016	One site	Retrospective	Cross-sectional	May/2013-May/2014	Tanzania	381	Moderate	Consecutive	Blood
**Kajeguka**	Febrile	2016	One site	Retrospective	Cross-sectional	May/2013-May/2014	Tanzania	622	Moderate	Consecutive	Blood
**Kolawole**	Febrile	2017	One site	Prospective	Cross-sectional	Jan/2016-Feb/2016	Nigeria	95	Low	Consecutive	Blood
**Konongoi**	Febrile	2016	One site	Prospective	Cross-sectional	Sep/2011-Dec/2014	Kenya	868	Low	Consecutive	Blood
**Laval**	Febrile	2013	Multisite	Prospective	Cross-sectional	Jan/2010-Dec/2011	Côte d'Ivoire	972	Moderate	Consecutive	Blood
**Laval**	Febrile	2013	Multisite	Prospective	Cross-sectional	Jan/2010-Dec/2011	Djibouti	2882	Moderate	Consecutive	Blood
**Laval**	Febrile	2013	Multisite	Prospective	Cross-sectional	Jan/2010-Dec/2011	Tanzania	1217	Moderate	Consecutive	Blood
**L'Azou**	Febrile	2015	One site	Prospective	Cross-sectional	Dec/2011-Dec2012	Côte d'Ivoire	796	Moderate	Consecutive	Blood
**Le Gonidec**	Febrile	2016	One site	Retrospective	Cross-sectional	Jan/2011-May/2014	Djibouti	354	Moderate	Consecutive	Blood
**Lobo**	Febrile	2016	One site	Prospective	Cross-sectional	Not defined	Sierra Leone	149	Low	Consecutive	Blood
**Makiala-M.**	Febrile	2018	One site	Prospective	Cross-sectional	Jan/2003-Jan/2012	D.R. of the Congo	423	Moderate	Consecutive	Blood
**Mazaba-Liwewe**	Apparently healthy	2014	One site	Prospective	Cross-sectional	Not defined	Zambia	3624	Low	Cluster	Blood
**Mease**	Apparently healthy	2011	One site	Prospective	Cross-sectional	Jul/2014-Sep/2014	Kenya	1141	Low	Consecutive	Blood
**Mustapha**	Febrile	2017	One site	Prospective	Cross-sectional	Not defined	Nigeria	178	Low	Consecutive	Blood
**Narkwa**	Apparently healthy	2016	One site	Prospective	Cross-sectional	Feb/2013-Dec/2015	Ghana	188	Low	Consecutive	Blood
**Nasira**	Febrile	2017	One site	Prospective	Cross-sectional	May/2016-Aug/2016	Nigeria	171	Low	Consecutive	Blood
**Ngoi**	Febrile	2016	One site	Prospective	Cross-sectional	2014-2015	Kenya	489	Low	Consecutive	Blood
**Nkoghe**	Febrile	2012	One site	Prospective	Cross-sectional	2010	Gabon	433	Moderate	Consecutive	Blood
**Noden**	Apparently healthy	2014	One site	Prospective	Cross-sectional	Sep/2011-Feb/2012	Namibia	312	Low	Consecutive	Blood
**Ochieng**	Apparently healthy	2015	One site	Prospective	Cross-sectional	2007	Kenya	1091	Low	Consecutive	Blood
**Oladipo**	Apparently healthy	2014	One site	Prospective	Cross-sectional	Not defined	Nigeria	93	Low	Consecutive	Blood
**Oludele**	Febrile	2017	One site	Prospective	Cross-sectional	Jan/2015-Mar/2016	Zimbabwe	192	Moderate	Consecutive	Blood
**Olufisayo**	Febrile	2016	One site	Prospective	Cross-sectional	Not defined	Nigeria	179	Low	Consecutive	Blood

**Onoja**	Febrile	2016	One site	Prospective	Cross-sectional	Apr/2014-Dec/2014	Nigeria	274	Low	Consecutive	Blood
**Otu**	Febrile	2019	One site	Prospective	Cross-sectional	Jan/2017-Aug/2017	Nigeria	400	Moderate	Consecutive	Blood
**Oyero**	Febrile	2014	One site	Prospective	Cross-sectional	Jan/2013-Apr/2013	Nigeria	188	Low	Consecutive	Blood
**Phoutrides**	Febrile	2011	One site	Retrospective	Cross-sectional	Jul/2006-	Mali	95	Moderate	Consecutive	Blood
**Ridde**	Febrile	2016	One site	Prospective	Cross-sectional	Dec/2013-Jan/2014	Burkina Faso	379	Moderate	Consecutive	Blood
**Sado**	Febrile	2018	One site	Prospective	Cross-sectional	Mar-Apr/2017	Cameroon	114	Low	Consecutive	Blood
**Schoepp**	Febrile	2014	One site	Retrospective	Cross-sectional	Oct/2006–Oct/2008	Sierra Leone	253	Low	Consecutive	Blood
**Seidahmed**	Apparently healthy	2012	One site	Prospective	Cross-sectional	Nov/2008-Oct/2009	Sudan	791	Low	Consecutive	Blood
**Sissoko**	Apparently healthy	2010	One site	Retrospective	Cross-sectional	Nov/2016-Dec/2006	Mayotte	1154	Low	Cluster	Blood
**Soghaier**	Apparently healthy	2015	One site	Prospective	Cross-sectional	Not defined	Sudan	491	Low	Cluster	Blood
**Soghaier**	Apparently healthy	2014	One site	Prospective	Cross-sectional	Not defined	Sudan	600	Low	Cluster	Blood
**Sow**	Febrile	2016	One site	Prospective	Cross-sectional	Jul2009-Mar2013	Senegal	13845	Moderate	Consecutive	Blood
**Stoler**	Febrile	2015	One site	Retrospective	Cross-sectional	2011-2014	Ghana	218	Low	Consecutive	Blood
**Tchuandom**	Febrile	2018	One site	Prospective	Cross-sectional	Mar/2016-Apr/2017	Cameroon	384	Low	Random	Blood
**Tsegaye**	Apparently healthy	2018	One site	Prospective	Cross-sectional	Not defined	Ethiopia	1645	Moderate	Consecutive	Blood
**Vairo**	Febrile	2012	One site	Prospective	Cross-sectional	Feb/2007-Mar/2007	Tanzania	202	Low	Consecutive	Blood
**Vairo**	Febrile	2017	One site	Prospective	Cross-sectional	Not defined	Tanzania	483	Low	Consecutive	Blood
**Vairo**	Apparently healthy	2014	One site	Prospective	Cross-sectional	Sep/2011-Dec/2011	Tanzania	500	Low	Consecutive	Blood
**Vu**	Apparently healthy	2016	One site	Prospective	Cross-sectional	2009-2011	Kenya	1863	Low	Cluster	Blood
**Woyessa**	Febrile	2013	One site	Prospective	Cross-sectional	Sep/2013-Dec/2013	Ethiopia	11409	Low	Consecutive	Blood
**Yen**	Apparently healthy	2016	One site	Prospective	Cross-sectional	2003-2004	Sao Tome and Principe	78	Low	Consecutive	Blood

NR: not reported

Supplementary Figure 1 : Funnel plot for publication in the IgG seroprevalence of Dengue virus infection in febrile participants in Africa

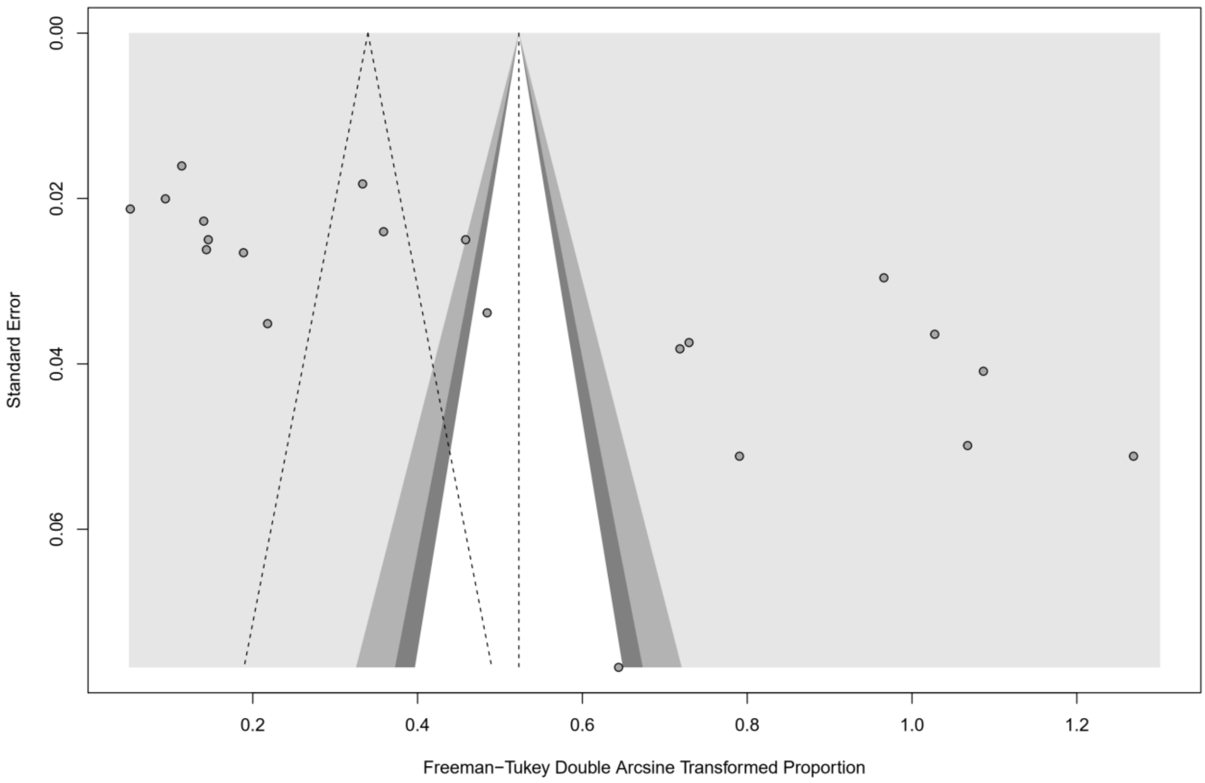

Supplementary Figure 2 : Funnel plot for publication in the IgG seroprevalence of Dengue virus infection in apparently healthy participants in Africa

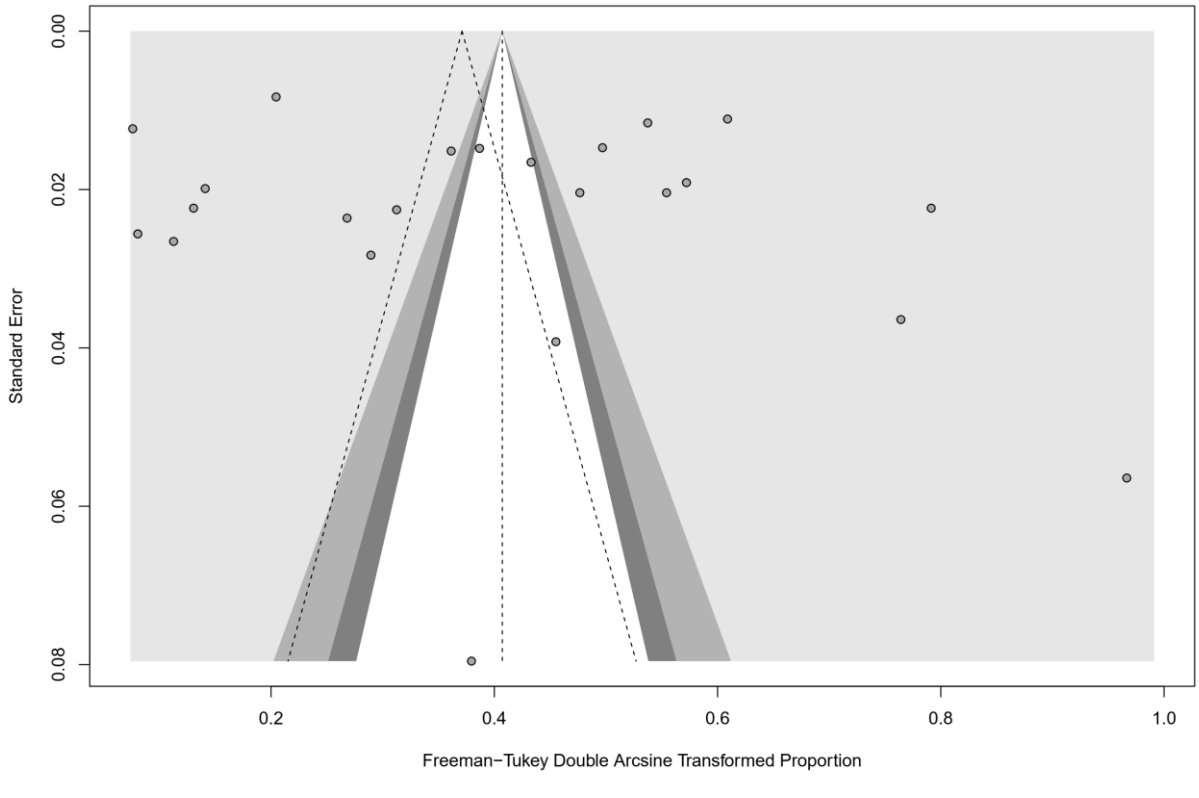

Supplementary Figure 3 : Funnel plot for publication in the IgM seroprevalence of Dengue virus infection in febrile participants in Africa

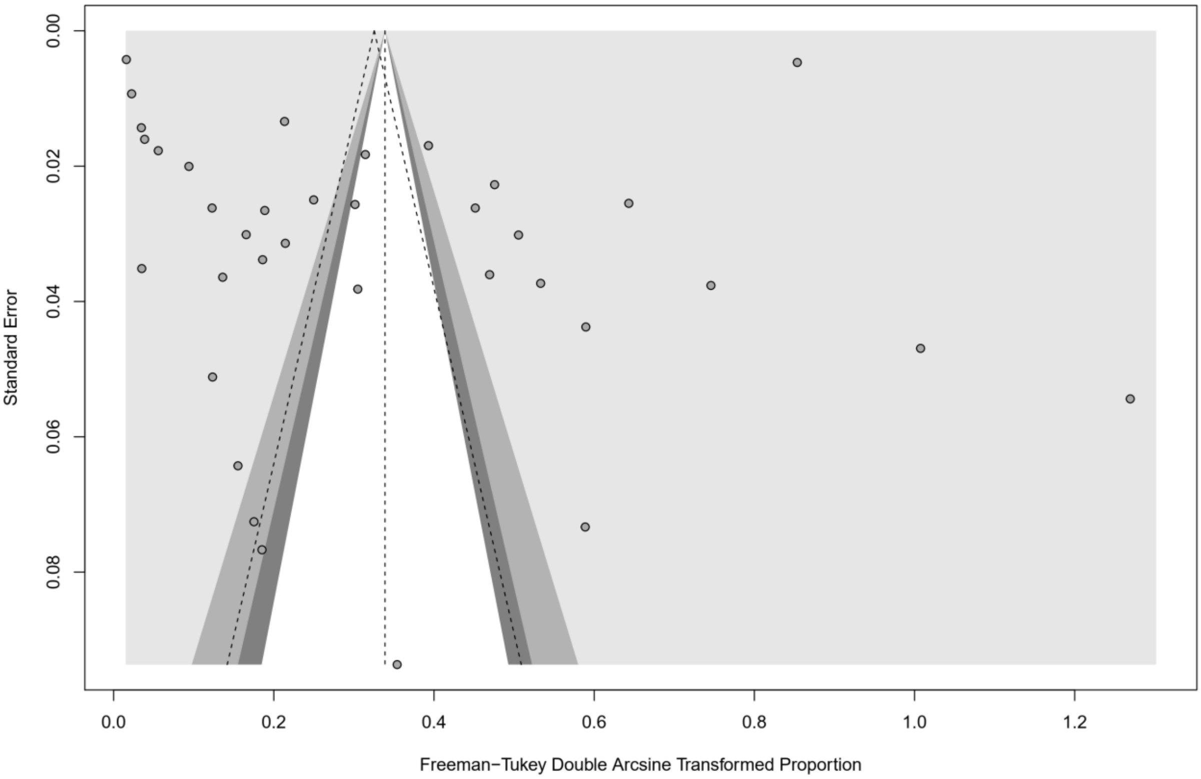

Supplementary Figure 4 : Funnel plot for publication in the IgM seroprevalence of Dengue virus infection in apparently healthy participants in Africa

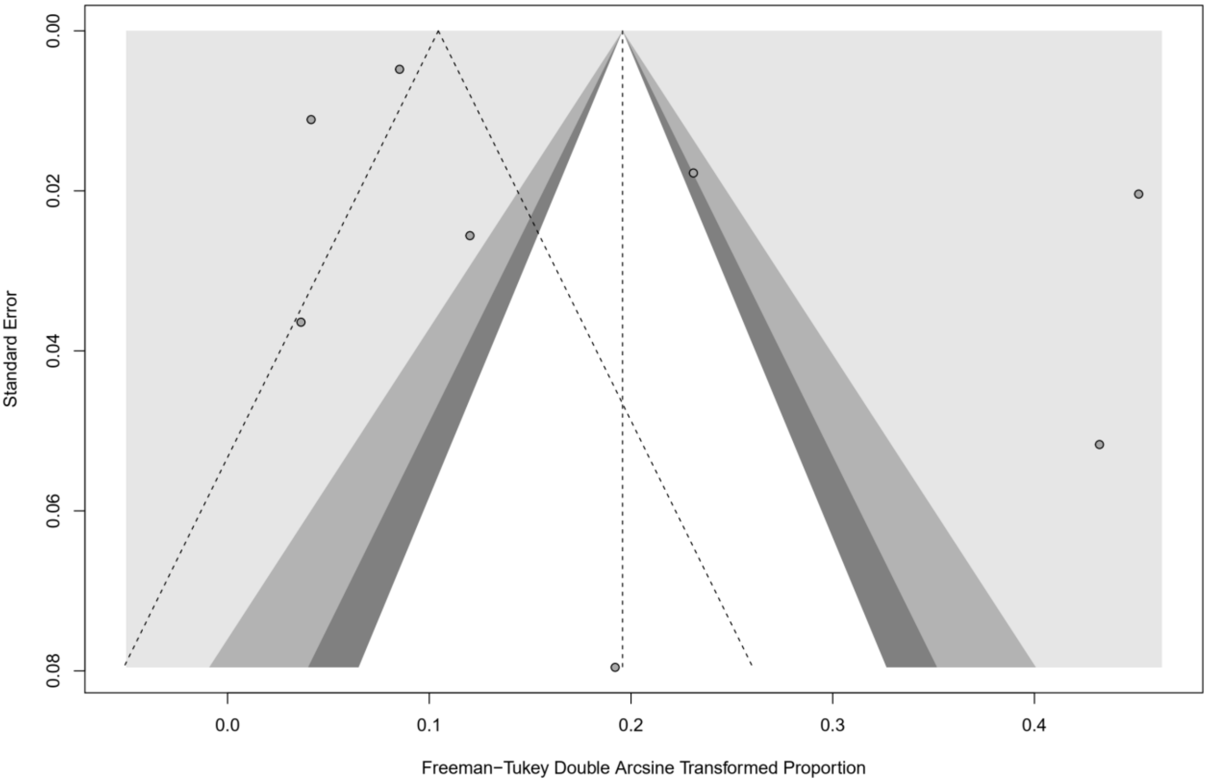

Supplementary Figure 5 : Funnel plot for publication in the RNA prevalence of Dengue virus infection in febrile participants in Africa

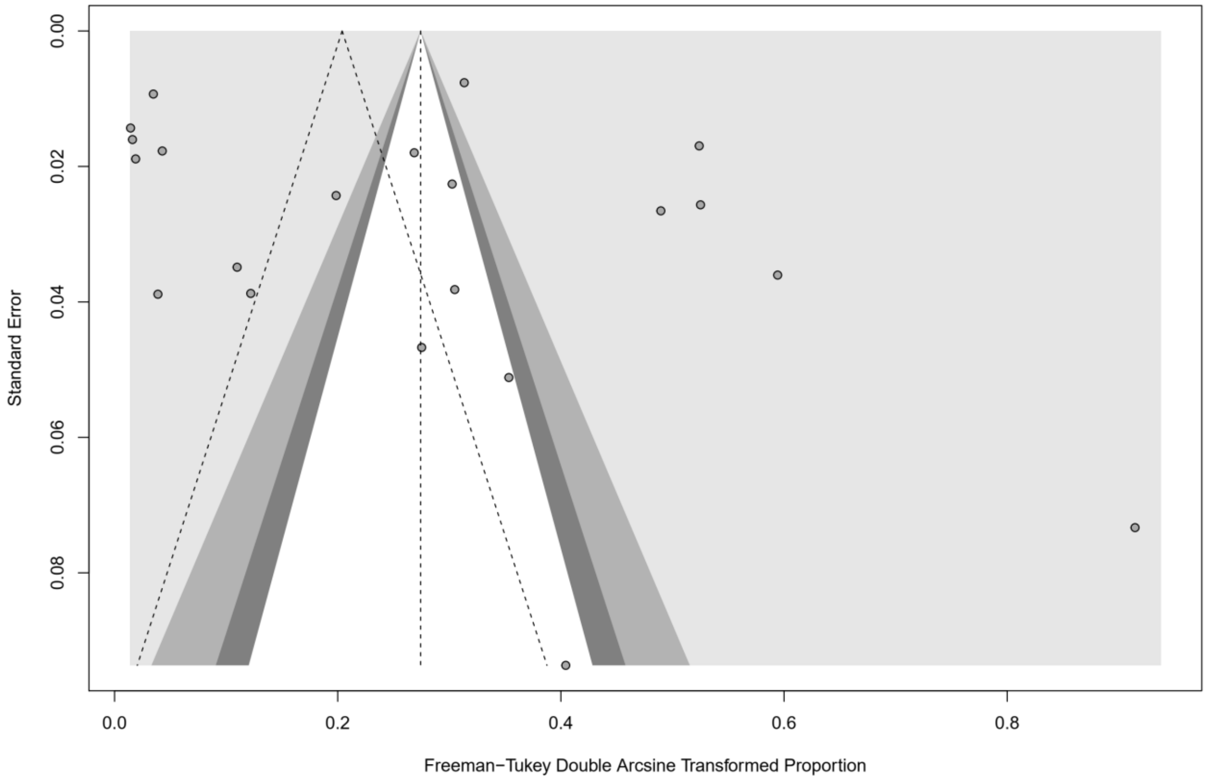

Supplementary Figure 6 : Forest plot IgG seroprevalence of Dengue virus infection in febrile participants in Africa, by region

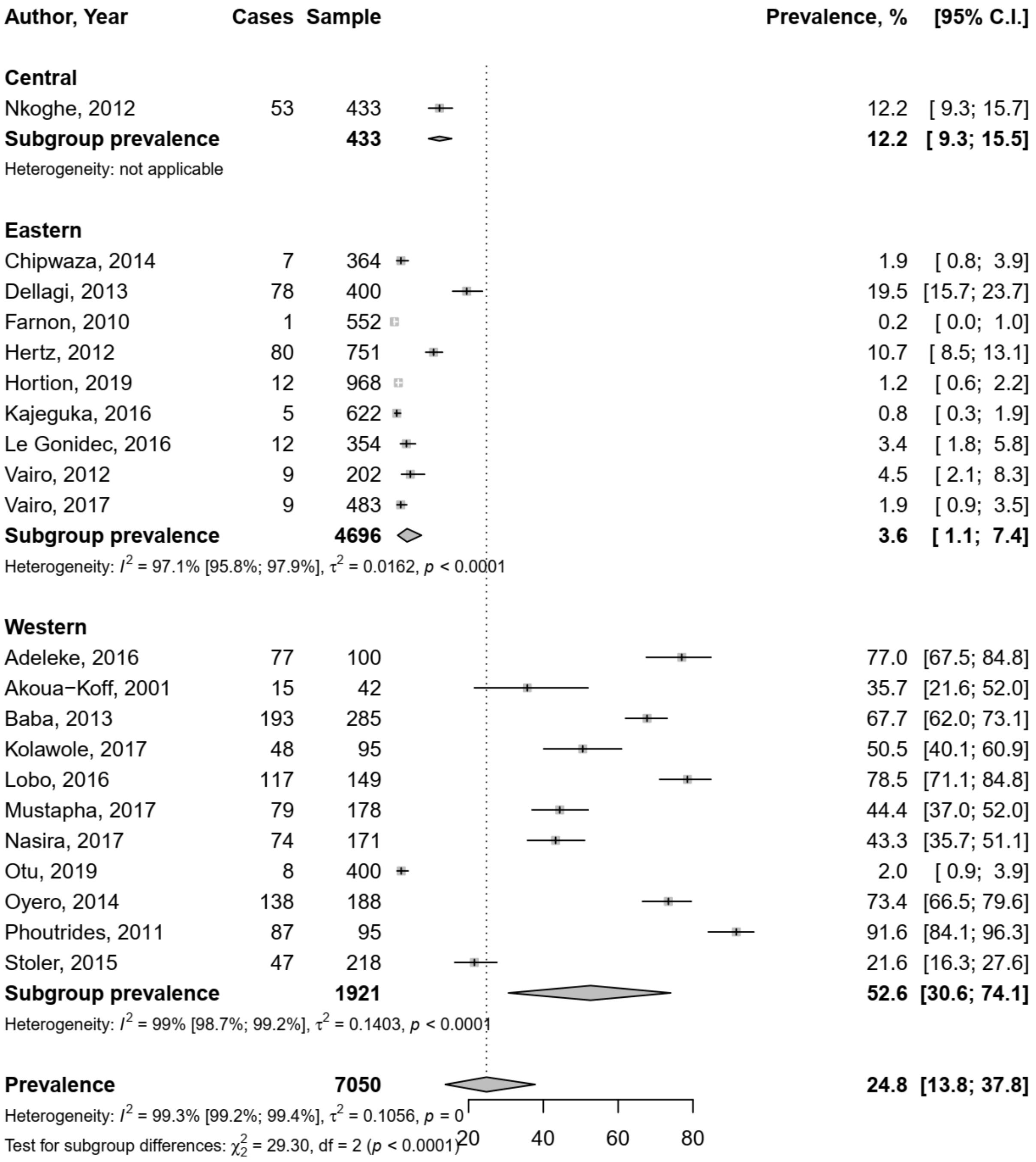

Supplementary Figure 7 : Forest plot IgG seroprevalence of Dengue virus infection in apparently healthy participants in Africa, by region

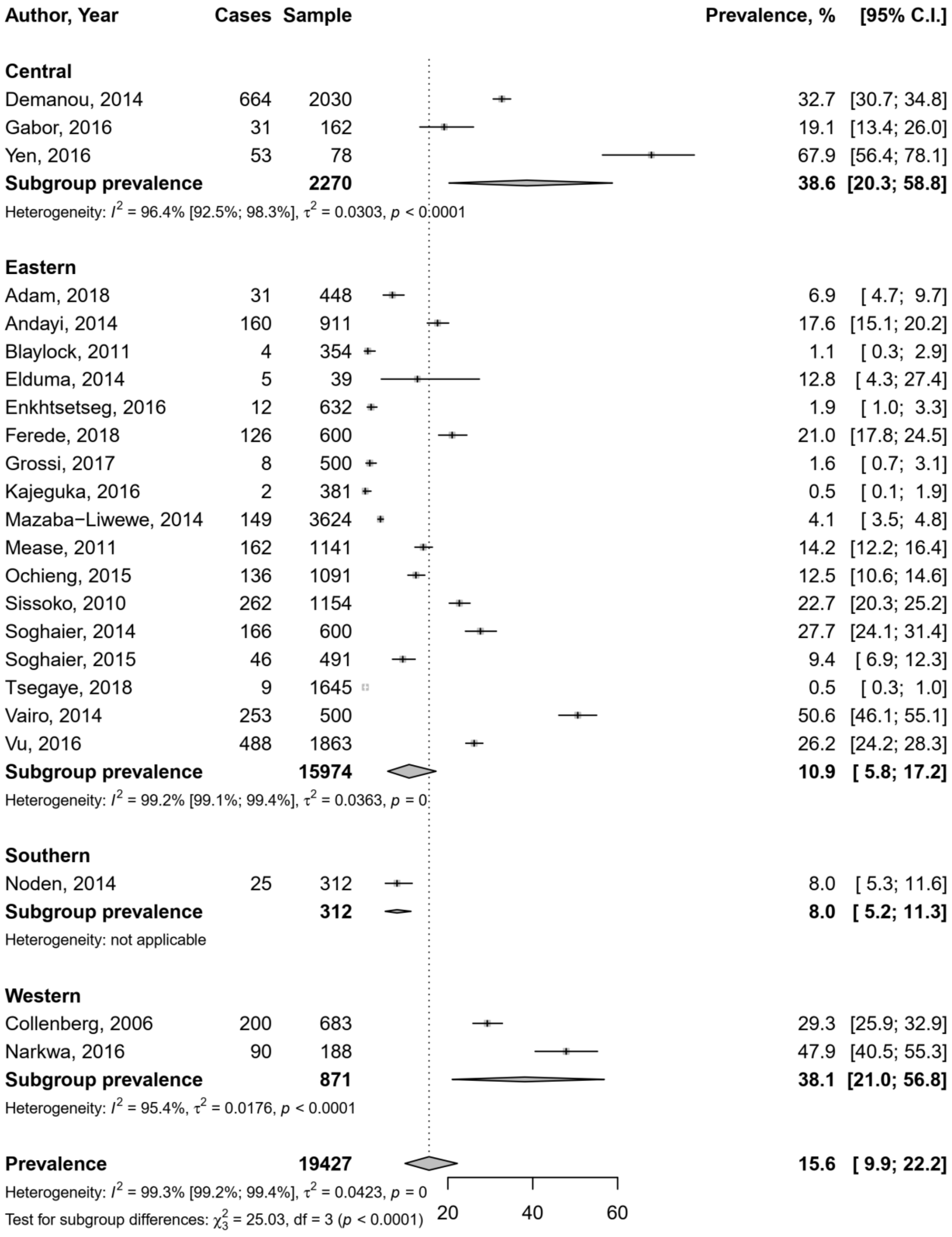

Supplementary Figure 8 : Forest plot IgM seroprevalence of Dengue virus infection in febrile participants in Africa, by region

Author, Year	Cases	Sample	Prevalence, %	[95% C.I.]
**Central**				
Hunsperger, 2016	14	46	30.4	[17.7; 45.8]
Tchuandom, 2018	138	384	35.9	[31.1; 41.0]
**Subgroup prevalence**		**430**	**35.2**	**[30.7; 39.9]**
Heterogeneity: $I^2 = 0\%$, $\tau^2 = 0$, $p = 0.4835$				
**Eastern**				
Abdalla, 2014	81	113	71.7	[62.4; 79.8]
Abdallah, 2014	7	275	2.5	[1.0; 5.2]
Ageep, 2006	77	84	91.7	[83.6; 96.6]
Chipwaza, 2014	69	364	19.0	[15.1; 23.4]
Dellagi, 2013	5	364	1.4	[0.4; 3.2]
Hertz, 2012	71	747	9.5	[7.5; 11.8]
Kajeguka, 2016	5	622	0.8	[0.3; 1.9]
Konongoi, 2016	127	868	14.6	[12.3; 17.2]
Laval, 2013	1	2882	0.0	[0.0; 0.2]
Laval, 2013	1	1217	0.1	[0.0; 0.5]
Le Gonidec, 2016	12	354	3.4	[1.8; 5.8]
Oludele, 2017	39	192	20.3	[14.9; 26.7]
Vairo, 2012	0	202	0.0	[0.0; 1.8]
Vairo, 2017	101	483	20.9	[17.4; 24.8]
Woyessa, 2013	6480	11409	56.8	[55.9; 57.7]
**Subgroup prevalence**		**20176**	**14.5**	**[2.5; 33.7]**
Heterogeneity: $I^2 = 99.9\%$ [99.9%; 99.9%], $\tau^2 = 0.2015$, $p = 0$				
**Western**				
Adedayo, 2013	40	130	30.8	[23.0; 39.5]
Akoua−Koff, 2001	1	42	2.4	[0.1; 12.6]
Aoussi, 2014	3	28	10.7	[2.3; 28.2]
Ariyonde, 2016	1	60	1.7	[0.0; 8.9]
Dariano, 2017	62	1392	4.5	[3.4; 5.7]
Jentes, 2010	1	47	2.1	[0.1; 11.3]
Kolawole, 2017	81	176	46.0	[38.5; 53.7]
L'Azou, 2015	2	796	0.3	[0.0; 0.9]
Laval, 2013	1	972	0.1	[0.0; 0.6]
Nasira, 2017	15	171	8.8	[5.0; 14.1]
Olufisayo, 2016	46	179	25.7	[19.5; 32.8]
Onoja, 2016	64	274	23.4	[18.5; 28.8]
Otu, 2019	24	400	6.0	[3.9; 8.8]
Oyero, 2014	3	188	1.6	[0.3; 4.6]
Phoutrides, 2011	1	95	1.1	[0.0; 5.7]
Ridde, 2016	33	379	8.7	[6.1; 12.0]
Schoepp, 2014	11	253	4.3	[2.2; 7.6]
Sow, 2016	3	13845	0.0	[0.0; 0.1]
Stoler, 2015	7	218	3.2	[1.3; 6.5]
**Subgroup prevalence**		**19645**	**6.6**	**[2.9; 11.6]**
Heterogeneity: $I^2 = 98.6\%$ [98.4%; 98.9%], $\tau^2 = 0.0338$, $p < 0.0001$				
**Prevalence**		**40251**	**10.8**	**[3.8; 20.6]**
Heterogeneity: $I^2 = 99.8\%$ [99.8%; 99.8%], $\tau^2 = 0.1688$, $p = 0$				
Test for subgroup differences: $\chi^2_2 = 57.55$, df = 2 ($p < 0.0001$)				

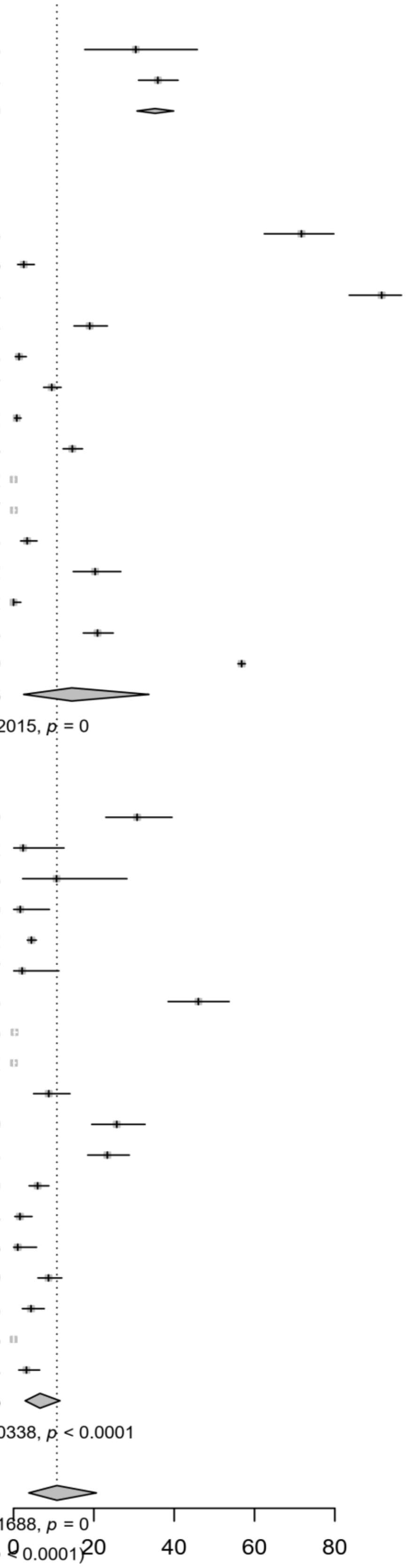

Supplementary Figure 9 : Forest plot IgM seroprevalence of Dengue virus infection in apparently healthy participants in Africa, by region

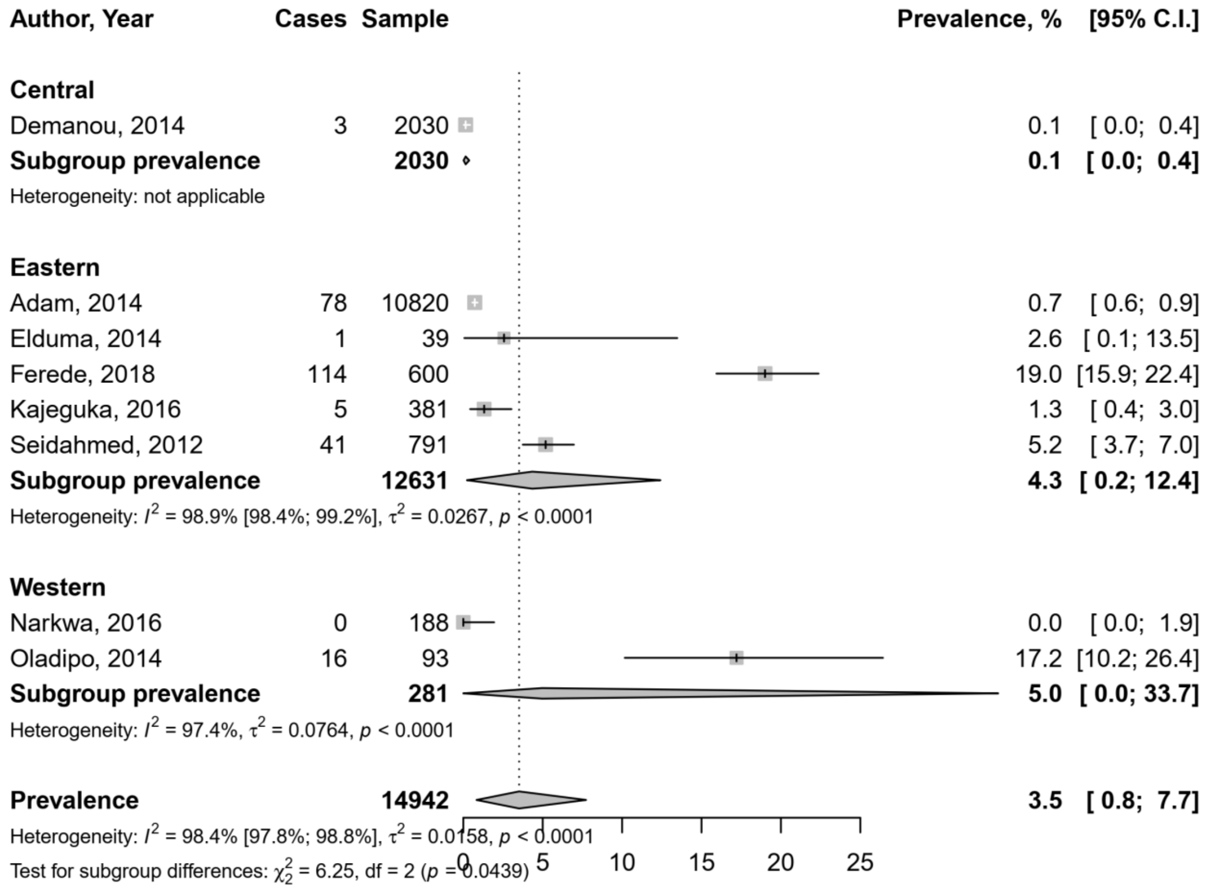

Supplementary Figure 10 : Forest plot dengue virus RNA prevalence of Dengue virus infection in febrile participants in Africa, by region

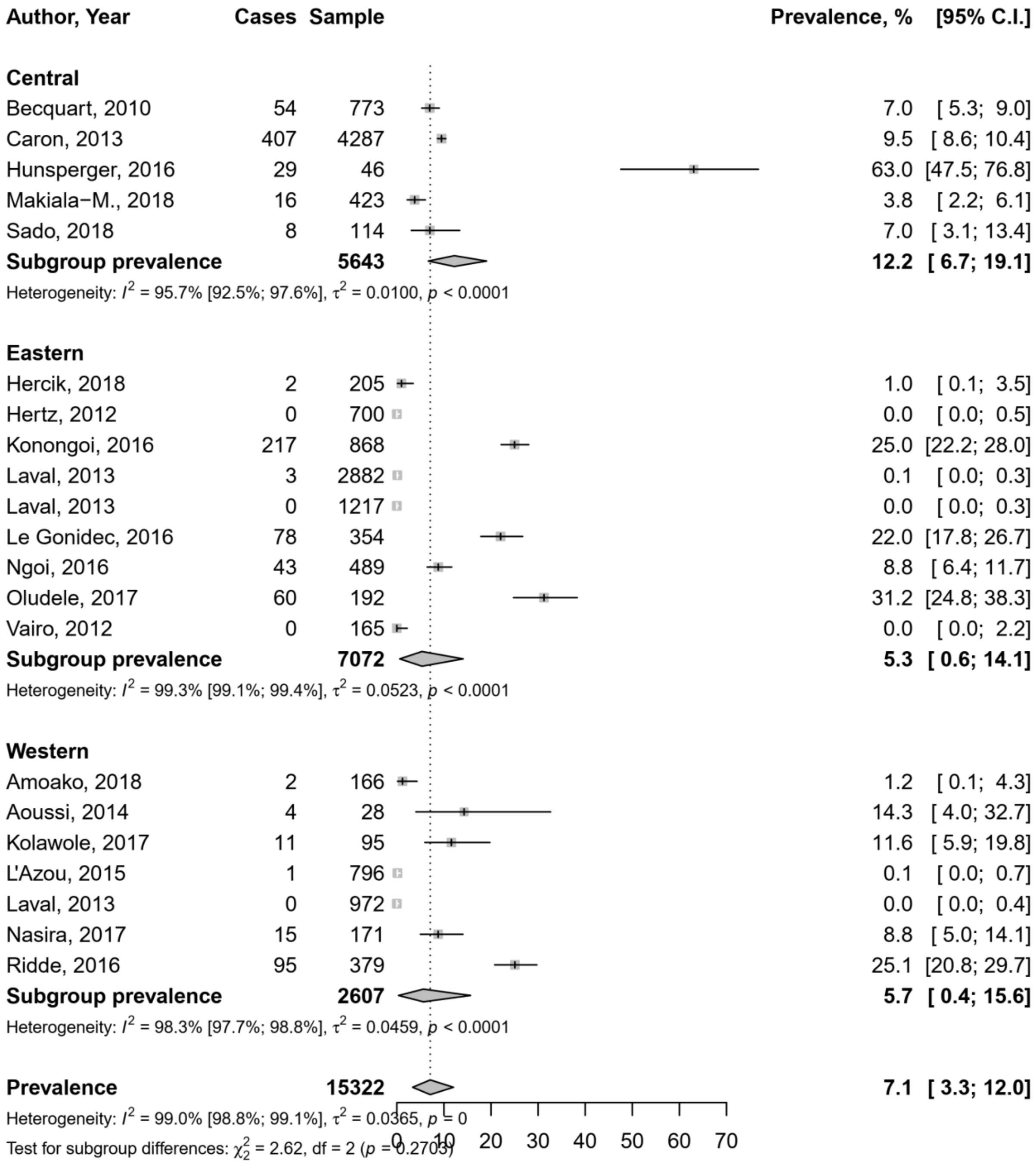

List of included studies[1-77]

1 Abdallah, T. M., Ali, A. A. A., Karsany, M. S. & Adam, I. Epidemiology of dengue infections in Kassala, Eastern Sudan. *Journal of Medical Virology* **84**, 500-503, doi:10.1002/jmv.23218 (2012).

2 Abdalla, T. M., Karsany, M. S. & Ali, A. A. Correlation of measles and dengue infection in Kassala, Eastern Sudan. *Journal of Medical Virology* **87**, 76-78, doi:10.1002/jmv.24001 (2015).

3 Adam, I., Jumaa, A. M., Elbashir, H. M. & Karsany, M. S. Maternal and perinatal outcomes of dengue in PortSudan, Eastern Sudan. *Virology Journal* **7**, doi:10.1186/1743-422X-7-153 (2010).

4 Adedayo, F., Nioma, I., Olanrewaju, M. B., Adeyinka, A. & Ebele, A. Serological evidence of recent dengue virus infection among febrile children in a semi arid zone. *American Journal of Infectious Diseases* **9**, 7-10, doi:10.3844/ajidsp.2013.7.10 (2013).

5 Adeleke, M. A. *et al.* Dengue virus specific Immunoglobulin G antibodies among patients with febrile conditions in Osogbo, Southwestern Nigeria. *Tropical Biomedicine* **33**, 1-7 (2016).

6 Ageep, A. K., Malik, A. A. & Elkarsani, M. S. Clinical presentations and laboratory findings in suspected cases of dengue virus. *Saudi Medical Journal* **27**, 1711-1713 (2006).

7 Akoua-Koffi, C. *et al.* Investigation surrounding a fatal case of yellow fever in Côte d'Ivoire in 1999. *Bulletin de la Société de pathologie exotique (1990)* **94**, 227-230 (2001).

8 Aoussi, E. B. F. *et al.* Seven native cases of dengue in Abidjan, Ivory Coast. *Medecine Et Maladies Infectieuses* **44**, 433-436, doi:10.1016/j.medmal.2014.08.002 (2014).

9 Andayi, F. *et al.* A Sero-epidemiological Study of Arboviral Fevers in Djibouti, Horn of Africa. *PLoS Neglected Tropical Diseases* **8**, doi:10.1371/journal.pntd.0003299 (2014).

10 Ayorinde, A. F., Oyeyiga, A. M., Nosegbe, N. O. & Folarin, O. A. A survey of malaria and some arboviral infections among suspected febrile patients visiting a health centre in Simawa, Ogun State, Nigeria. *Journal of Infection and Public Health* **9**, 52-59, doi:10.1016/j.jiph.2015.06.009 (2016).

11 Baba, M. *et al.* Evidence of arbovirus co-infection in suspected febrile malaria and typhoid patients in Nigeria. *Journal of Infection in Developing Countries* **7**, 51-59 (2013).

12 Babaniyi, O. *et al.* Risk assessment for yellow fever in western and North-Western provinces of Zambia. *Journal of Global Infectious Diseases* **7**, 11-17, doi:10.4103/0974-777X.150884 (2015).

13 Becquart, P. *et al.* Acute dengue virus 2 infection in Gabonese patients is associated with an early innate immune response, including strong interferon alpha production. *BMC Infectious Diseases* **10**, doi:10.1186/1471-2334-10-356 (2010).

14 Blaylock, J. M. *et al.* The seroprevalence and seroincidence of dengue virus infection in western Kenya. *Travel Medicine and Infectious Disease* **9**, 246-248, doi:10.1016/j.tmaid.2011.06.005 (2011).

15 Caron, M. *et al.* First Evidence of Simultaneous Circulation of Three Different Dengue Virus Serotypes in Africa. *PLoS ONE* **8**, doi:10.1371/journal.pone.0078030 (2013).

16 Chipwaza, B. *et al.* Dengue and Chikungunya Fever among Viral Diseases in Outpatient Febrile Children in Kilosa District Hospital, Tanzania. *PLoS Neglected Tropical Diseases* **8**, doi:10.1371/journal.pntd.0003335 (2014).

17 Collenberg, E. *et al.* Seroprevalence of six different viruses among pregnant women and blood donors in rural and urban Burkina Faso: A comparative analysis. *Journal of Medical Virology* **78**, 683-692, doi:10.1002/jmv.20593 (2006).

18 Dariano, D. F. *et al.* Surveillance of vector-borne infections (chikungunya, dengue, and malaria) in Bo, Sierra Leone, 2012-2013. *American Journal of Tropical Medicine and Hygiene* **97**, 1151-1154, doi:10.4269/ajtmh.16-0798 (2017).

19 Dellagi, K. *et al.* Serological Evidence of Contrasted Exposure to Arboviral Infections between Islands of the Union of Comoros (Indian Ocean). *Plos Neglected Tropical Diseases* **10**, doi:10.1371/journal.pntd.0004840 (2016).

20 Demanou, M. *et al.* Evidence of Dengue Virus Transmission and Factors Associated with the Presence of Anti-Dengue Virus Antibodies in Humans in Three Major Towns in Cameroon. *Plos Neglected Tropical Diseases* **8**, doi:10.1371/journal.pntd.0002950 (2014).

21 Elduma, A. H. & Osman, W. M. Dengue and hepatitis E virus infection in pregnant women in Eastern Sudan, A challenge for diagnosis in an endemic area. *Pan African Medical Journal* **19**, doi:10.11604/pamj.2014.19.391.5439 (2014).

22 Enkhtsetseg, A. *et al.* Seroconversion to causes of febrile illness in Mongolian peacekeepers deployed to south Sudan. *American Journal of Tropical Medicine and Hygiene* **95**, 1469-1471, doi:10.4269/ajtmh.16-0174 (2016).

23 Farnon, E. C. *et al.* Household-Based Sero-Epidemiologic Survey after a Yellow Fever Epidemic, Sudan, 2005. *American Journal of Tropical Medicine and Hygiene* **82**, 1146-1152, doi:10.4269/ajtmh.2010.09-0105 (2010).

24 Fokam, E. B. *et al.* Silent circulation of arboviruses in Cameroon. *East African medical journal* **87**, 262-268 (2010).

25 Gabor, J. J., Schwarz, N. G., Esen, M., Kremsner, P. G. & Grobusch, M. P. Dengue and chikungunya seroprevalence in Gabonese infants prior to major outbreaks in 2007 and 2010: A sero-epidemiological study. *Travel Medicine and Infectious Disease* **14**, 26-31, doi:10.1016/j.tmaid.2016.01.005 (2016).

26 Grossi-Soyster, E. N. *et al.* Serological and spatial analysis of alphavirus and flavivirus prevalence and risk factors in a rural community in western Kenya. *Plos Neglected Tropical Diseases* **11**, doi:10.1371/journal.pntd.0005998 (2017).

27 Hertz, J. T. *et al.* Chikungunya and dengue fever among hospitalized febrile patients in Northern Tanzania. *American Journal of Tropical Medicine and Hygiene* **86**, 171-177, doi:10.4269/ajtmh.2012.11-0393 (2012).

28 Hunsperger, E. A. *et al.* Use of a rapid test for diagnosis of dengue during suspected dengue outbreaks in resource-limited regions. *Journal of Clinical Microbiology* **54**, 2090-2095, doi:10.1128/JCM.00521-16 (2016).

29 Jentes, E. S. *et al.* Acute arboviral infections in Guinea, West Africa, 2006. *American Journal of Tropical Medicine and Hygiene* **83**, 388-394, doi:10.4269/ajtmh.2010.09-0688 (2010).

30 Kajeguka, D. C. *et al.* Prevalence of dengue and chikungunya virus infections in north-eastern Tanzania: a cross sectional study among participants presenting with malaria-like symptoms. *Bmc Infectious Diseases* **16**, doi:10.1186/s12879-016-1511-5 (2016).

31 Kolawole, O. M., Seriki, A. A., Irekeola, A. A., Bello, K. E. & Adeyemi, O. O. Dengue virus and malaria concurrent infection among febrile subjects within Ilorin metropolis, Nigeria. *Journal of Medical Virology* **89**, 1347-1353, doi:10.1002/jmv.24788 (2017).

32 Konongoi, L. *et al.* Detection of dengue virus serotypes 1, 2 and 3 in selected regions of Kenya: 2011-2014. *Virology Journal* **13**, 1-11, doi:10.1186/s12985-016-0641-0 (2016).

33 Kuniholm, M. H. *et al.* Seroprevalence and distribution of Flaviviridae, Togaviridae, and Bunyaviridae arboviral infections in rural Cameroonian adults. *American Journal of Tropical Medicine and Hygiene* **74**, 1078-1083 (2006).

34 de Laval, F. *et al.* Dengue Surveillance in the French Armed Forces: A Dengue Sentinel Surveillance System in Countries Without Efficient Local Epidemiological Surveillance. *Journal of Travel Medicine* **20**, 259-261, doi:10.1111/j.1708-8305.2012.00674.x (2013).

35 L'Azou, M. *et al.* Dengue: etiology of acute febrile illness in Abidjan, Cote d'Ivoire, in 2011-2012. *Transactions of the Royal Society of Tropical Medicine and Hygiene* **109**, 717-722, doi:10.1093/trstmh/trv076 (2015).

36 Le Gonidec, E. *et al.* Clinical Survey of Dengue Virus Circulation in the Republic of Djibouti between 2011 and 2014 Identifies Serotype 3 Epidemic and Recommends Clinical Diagnosis Guidelines for Resource Limited Settings. *Plos Neglected Tropical Diseases* **10**, doi:10.1371/journal.pntd.0004755 (2016).

37 Lobo, J. M. D., Mores, C. N., Bausch, D. G. & Christofferson, R. C. Short Report: Serological Evidence of Under-Reported Dengue Circulation in Sierra Leone. *Plos Neglected Tropical Diseases* **10**, doi:10.1371/journal.pntd.0004613 (2016).

38 Mazaba-Liwewe, M. L. *et al.* First sero-prevalence of dengue fever specific immunoglobulin G antibodies in Western and North-Western provinces of Zambia: A population based cross sectional study. *Virology Journal* **11**, doi:10.1186/1743-422X-11-135 (2014).
39 Mease, L. E. *et al.* Seroprevalence and distribution of arboviral infections among rural Kenyan adults: A cross-sectional study. *Virology Journal* **8**, doi:10.1186/1743-422X-8-371 (2011).
40 Mustapha, J. O., Emeribe, A. U. & Nasir, I. A. Survey of malaria and anti-dengue virus IgG among febrile HIV-infected patients attending a tertiary hospital in Abuja, Nigeria. *HIV/AIDS - Research and Palliative Care* **9**, 145-151, doi:10.2147/HIV.S134023 (2017).
41 Narkwa, P. W. *et al.* Dengue virus exposure among blood donors in Ghana. *Journal of Medical and Biomedical Sciences* **5**, 30-35, doi:10.4314/jmbs.v5i2.5 (2016).
42 Nasira, I. A., Agbede, O. O., Dangana, A., Baba, M. & Haruna, A. S. Dengue virus non-structural Protein-1 expression and associated risk factors among febrile Patients attending University of Abuja Teaching Hospital, Nigeria. *Virus Research* **230**, 7-12, doi:10.1016/j.virusres.2016.12.011 (2017).
43 Ngoi, C. N. *et al.* Dengue and chikungunya virus infections among young febrile adults evaluated for acute HIV-1 infection in coastal Kenya. *PLoS ONE* **11**, doi:10.1371/journal.pone.0167508 (2016).
44 Nkoghe, D. *et al.* No clinical or biological difference between Chikungunya and Dengue fever during the 2010 Gabonese outbreak. *Infectious Disease Reports* **4**, 11-13, doi:10.4081/idr.2012.e05 (2012).
45 Noden, B. H. *et al.* Risk assessment of flavivirus transmission in Namibia. *Acta Tropica* **137**, 123-129, doi:10.1016/j.actatropica.2014.05.010 (2014).
46 Ochieng, C. *et al.* Seroprevalence of infections with dengue, Rift Valley fever and chikungunya viruses in Kenya, 2007. *PLoS ONE* **10**, doi:10.1371/journal.pone.0132645 (2015).
47 Oladipo, E. K., Amanetu, C., Gbadero, T. A. & Oloke, J. K. Detectable anti-dengue virus IgM antibodies among healthy individuals in Ogbomoso, Oyo state, Nigeria. *American Journal of Infectious Diseases* **10**, 64-67, doi:10.3844/ajidsp.2014.64.67 (2014).
48 Oludele, J. *et al.* Dengue virus serotype 2 established in northern Mozambique (2015–2016). *American Journal of Tropical Medicine and Hygiene* **97**, 1418-1422, doi:10.4269/ajtmh.17-0317 (2017).
49 Olufisayo, A. & Johnson, A. Incidence of dengue virus infections in febrile episodes in Ile-Ife, Nigeria. *African Journal of Infectious Diseases* **10**, 21-24, doi:10.4314/ajid.v10i1.4 (2016).
50 Onoja, A. B., Adeniji, J. A. & Olaleye, O. D. High rate of unrecognized dengue virus infection in parts of the rainforest region of Nigeria. *Acta Tropica* **160**, 39-43, doi:10.1016/j.actatropica.2016.04.007 (2016).
51 Oyero, O. G. & Ayukekbong, J. A. High dengue NS1 antigenemia in febrile patients in Ibadan, Nigeria. *Virus Research* **191**, 59-61, doi:10.1016/j.virusres.2014.07.023 (2014).
52 Phoutrides, E. K. *et al.* Dengue virus seroprevalence among febrile patients in Bamako, Mali: Results of a 2006 surveillance study. *Vector-Borne and Zoonotic Diseases* **11**, 1479-1485, doi:10.1089/vbz.2011.0622 (2011).
53 Ridde, V. *et al.* Presence of three dengue serotypes in Ouagadougou (Burkina Faso): research and public health implications. *Infectious Diseases of Poverty* **5**, doi:10.1186/s40249-016-0120-2 (2016).
54 Schoepp, R. J., Rossi, C. A., Khan, S. H., Goba, A. & Fair, J. N. Undiagnosed acute viral febrile illnesses, Sierra Leone. *Emerging Infectious Diseases* **20**, 1176-1182, doi:10.3201/eid2007.131265 (2014).
55 Seidahmed, O. M. E. *et al.* Spatial and Temporal Patterns of Dengue Transmission along a Red Sea Coastline: A Longitudinal Entomological and Serological Survey in Port Sudan City. *PLoS Neglected Tropical Diseases* **6**, doi:10.1371/journal.pntd.0001821 (2012).
56 Sissoko, D. *et al.* Seroepidemiology of Dengue Virus in Mayotte, Indian Ocean, 2006. *Plos One* **5**, doi:10.1371/journal.pone.0014141 (2010).
57 Soghaier, M. A. *et al.* Factors associated with dengue fever IgG sero-prevalence in South Kordofan State, Sudan, in 2012: Reporting prevalence ratios. *Journal of Infection and Public Health* **7**, 54-61, doi:10.1016/j.jiph.2013.07.008 (2014).

58 Soghaier, M. A. *et al.* Cross-sectional community-based study of the socio-demographic factors associated with the prevalence of dengue in the eastern part of Sudan in 2011. *BMC public health* **15**, 558, doi:10.1186/s12889-015-1913-0 (2015).
59 Sow, A. *et al.* Concurrent malaria and arbovirus infections in Kedougou, southeastern Senegal. *Malaria Journal* **15**, doi:10.1186/s12936-016-1100-5 (2016).
60 Stoler, J. *et al.* Evidence of Recent Dengue Exposure among Malaria Parasite-Positive Children in Three Urban Centers in Ghana. *American Journal of Tropical Medicine and Hygiene* **92**, 497-500, doi:10.4269/ajtmh.14-0678 (2015).
61 Sutherland, L. J. *et al.* Serologic evidence of arboviral infections among humans in Kenya. *American Journal of Tropical Medicine and Hygiene* **85**, 158-161, doi:10.4269/ajtmh.2011.10-0203 (2011).
62 Vairo, F. *et al.* IgG Against Dengue Virus in Healthy Blood Donors, Zanzibar, Tanzania. *Emerging Infectious Diseases* **20**, 465-468, doi:10.3201/eid2003.130150 (2014).
63 Vairo, F. *et al.* Seroprevalence of dengue infection: A cross-sectional survey in mainland Tanzania and on Pemba Island, Zanzibar. *International Journal of Infectious Diseases* **16**, e44-e46, doi:10.1016/j.ijid.2011.09.018 (2012).
64 Vairo, F. *et al.* Clinical, virologic, and epidemiologic characteristics of dengue outbreak, Dar es Salaam, Tanzania, 2014. *Emerging Infectious Diseases* **22**, 895-899, doi:10.3201/eid2205.151462 (2016).
65 Vu, D. M. *et al.* Dengue and west Nile virus transmission in children and adults in coastal Kenya. *American Journal of Tropical Medicine and Hygiene* **96**, 141-143, doi:10.4269/ajtmh.16-0562 (2017).
66 Woyessa, A. B. *et al.* The first acute febrile illness investigation associated with dengue fever in Ethiopia, 2013: A descriptive analysis. *Ethiopian Journal of Health Development* **28**, 155-161 (2014).
67 Yen, T. Y. *et al.* Seroprevalence of antibodies against dengue virus among pregnant women in the Democratic Republic of Sao Tome and Principe. *Acta Tropica* **155**, 58-62, doi:10.1016/j.actatropica.2015.12.012 (2016).
68 Makiala-Mandanda, S. *et al.* Identification of dengue and chikungunya cases among suspected cases of yellow fever in the democratic republic of the congo. *Vector-Borne and Zoonotic Diseases* **18**, 364-370, doi:10.1089/vbz.2017.2176 (2018).
69 Hortion, J. *et al.* Acute flavivirus and alphavirus infections among children in two different areas of Kenya, 2015. *American Journal of Tropical Medicine and Hygiene* **100**, 170-173, doi:10.4269/ajtmh.18-0297 (2019).
70 Hercik, C. *et al.* A combined syndromic approach to examine viral, bacterial, and parasitic agents among febrile patients: A pilot study in Kilombero, Tanzania. *American Journal of Tropical Medicine and Hygiene* **98**, 625-632, doi:10.4269/ajtmh.17-0421 (2018).
71 Mengesha Tsegaye, M. *et al.* Sero-prevalence of yellow fever and related Flavi viruses in Ethiopia: a public health perspective. *BMC Public Health* **18**, 1011, doi:10.1186/s12889-018-5726-9 (2018).
72 Otu, A. A. *et al.* A cross-sectional survey on the seroprevalence of dengue fever in febrile patients attending health facilities in Cross River State, Nigeria. *PLoS ONE* **14**, doi:10.1371/journal.pone.0215143 (2019).
73 Ferede, G. *et al.* A serologic study of dengue in northwest Ethiopia: Suggesting preventive and control measures. *PLoS Neglected Tropical Diseases* **12**, doi:10.1371/journal.pntd.0006430 (2018).
74 Yousseu, F. B. S., Nemg, F. B. S., Ngouanet, S. A., Mekanda, F. M. O. & Demanou, M. Detection and serotyping of dengue viruses in febrile patients consulting at the New-Bell District Hospital in Douala, Cameroon. *PLoS ONE* **13**, doi:10.1371/journal.pone.0204143 (2018).
75 Adam, A., Schüttoff, T., Reiche, S. & Jassoy, C. High seroprevalence of dengue virus indicates that dengue virus infections are frequent in central and eastern Sudan. *Tropical Medicine and International Health* **23**, 960-967, doi:10.1111/tmi.13116 (2018).

76 Tchuandom, S. B. *et al.* Seroprevalence of dengue virus among children presenting with febrile illness in some public health facilities in Cameroon. *The Pan African medical journal* **31**, 177, doi:10.11604/pamj.2018.31.177.16390 (2018).

77 Amoako, N. *et al.* Detection of dengue virus among children with suspected malaria, Accra, Ghana. *Emerging Infectious Diseases* **24**, 1561-1564, doi:10.3201/eid2408.180341 (2018).
